# Selective Chain Scission of L‐Lactide/ε‐Caprolactone Chain Shuttled Copolymers

**DOI:** 10.1002/cssc.71174

**Published:** 2026-10-30

**Authors:** A. Noon, L. Fichaux, X. Mosca, P. A. Lowy, T. Bousquet, L. Pelinski, F. Bonnet, J. P. P. Ramalho, A. Favrelle‐Huret, P. Zinck

**Affiliations:** ^1^ UCCS – Unité de Catalyse et Chimie du Solide (UMR 8181), Univ. Lille, CNRS, Centrale Lille, Univ. Artois Villeneuve d’Ascq France; ^2^ UMET – Unité Matériaux et Transformations (UMR 8207), Univ. Lille, CNRS, INRAE, Centrale Lille Lille France; ^3^ Chemistry and Biochemistry Department Hercules Centre Sciences and Technology School University of Évora Évora Portugal

**Keywords:** biopolyester degradation, multiblock copolymers, organocatalysis, selective degradation, transesterification

## Abstract

In this work, we investigated selective strategies for the degradation of L‐lactide/ε‐caprolactone chain shuttled multiblock copolymers composed of PLLA‐rich hard blocks and poly(ε‐caprolactone‐*co*‐L‐lactide) soft blocks. Enzymatic hydrolysis using the selective lipase from *Pseudomonas cepacia*, initially performed in aqueous buffer systems and subsequently in the presence of organic cosolvents, showed limited efficiency, likely due to limited enzymatic accessibility within the complex multiblock architecture. In contrast, homogeneous organocatalytic approaches showed higher degradation efficiency and selectivity. Base‐catalyzed transesterification using 4‐dimethylaminopyridine (DMAP) and imidazole enabled 100% selective chain scission toward lactyl units. Brønsted acid catalysis with p‐toluenesulfonic acid (pTSA) and diphenyl phosphate (DPP) exhibited complementary transesterification selectivity, favoring degradation of caprolactoyl units and thus targeting the soft segments. The latter selectivity is particularly interesting, as it provides structural information about the chain‐shuttled copolymers and allows to envision recycling of the PLA part. It is corroborated by DFT studies.

## Graphical abstract

L‐lactide/ε‐caprolactone chain shuttled multiblock copolymers are composed of PLLA‐rich hard blocks and poly(ε‐caprolactone‐*co*‐L‐lactide) soft blocks. They can be selectively degraded, notably into PLA oligomers, by organocatalyzed transesterification. This allows not only to determine the block length but also to envision the recycling of the PLA part into lactide monomer.

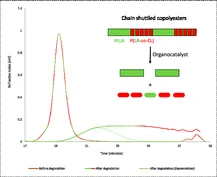

## 1 Introduction

The continuous growth in polymer production has led to a substantial increase in plastic waste, posing serious environmental concerns. Approximately 400 million tons of synthetic polymers are produced annually [1], yet only about 5%–6% are effectively recycled [2]. In Europe, plastics account for nearly 16% of municipal solid waste [3], underscoring the urgent need for sustainable waste management strategies. In this context, controlled degradation and advanced recycling processes play a crucial role in mitigating the environmental impact of polymeric materials and promoting circular material design. Biodegradable/compostable polymers have therefore emerged as promising alternatives for more sustainable material development [4, 5, 6, 7]. Among them, poly(lactide) (PLA) and poly(ε‐caprolactone) (PCL), as well as their blends and copolymers, are widely employed in industrial and biomedical applications due to their biodegradability, biocompatibility, and favorable physicochemical properties [8, 9, 10]. These polyesters also offer the possibility of hydrolysis [11] or accelerated and controlled depolymerization under tailored enzymatic or catalytic conditions [12, 13, 14, 15].

We recently reported the synthesis of multiblock copolymers consisting of alternating poly(L‐lactide) (PLLA)‐rich hard segments and poly(ε‐caprolactone‐*co*‐L‐lactide) statistical soft blocks [16, 17] . These materials were prepared via chain‐shuttling copolymerization (CSP) of L‐lactide and ε‐caprolactone, a one‐pot, one‐step strategy in which the growing polymer chains reversibly shuttle between two catalysts with different reactivity ratios. In the present work, we did investigate selective strategies for the degradation of these L‐lactide/ε‐caprolactone chain‐shuttled copolymers. Biodegradation of biocopolyesters might be of interest for biomedical applications. A selective degradation of one type of block could also help gain structural insight. Indeed, the number‐average molecular weight of the two types of blocks as well as the total number of blocks in the copolymer are currently unknown. They are not accessible via NMR studies, and the one‐pot character of CSP does not allow isolation of a part of the formed material for analysis, as done in conventional block copolymerization. We thus aim to develop selective degradation strategies that preferentially degrade the poly(ε‐caprolactone‐*co*‐L‐lactide) soft segments (Figure 1).

**FIGURE 1:**
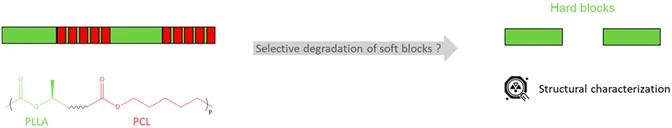
L‐lactide/ε‐caprolactone chain‐shuttled copolymers and aimed selective degradation of poly(ε‐caprolactone‐*co*‐L‐lactide) soft blocks enabling isolation of PLLA‐rich hard segments.

Enzymatic degradation represents an attractive strategy for polyester depolymerization under mild and environmentally friendly conditions while also providing inherent selectivity [18, 19, 20, 21]. A lipase from *Pseudomonas cepacia* was shown to efficiently degrade ε‐caprolactone‐rich poly(ε‐caprolactone‐*co*‐D,L‐lactide) statistical copolymers through ester bond hydrolysis in phosphate buffer, whereas PLA homopolymers and copolymers with high lactide content remained essentially unaffected [21]. This selective hydrolytic behavior might be relevant to the present study. Indeed, in the case of chain‐shuttled multiblock copolymers, composed of PLLA‐rich hard segments and poly(ε‐caprolactone‐*co*‐L‐lactide) soft domains, lipase selectivity toward caprolactoyl segments may offer the possibility of preferential cleavage of the soft blocks while preserving the PLLA‐rich hard segments. The same enzyme (*P. cepacia*) was also shown to afford selective degradation of PCL blocks in PCL‐poly(*p*‐dioxanone) (PPDO) multiblock copolymers [22]. In our case, owing to the structural complexity of the chain‐shuttled copolymers, enzymatic‐catalyzed degradation studies were extended beyond purely aqueous conditions to include organic solvent‐based systems capable of promoting polymer chain mobility [23] and enhancing polymer–enzyme interactions.

The structural complexity of the chain‐shuttled copolymers also motivated the investigation of alternative homogeneous degradation pathways in organic media using organocatalysts. Organocatalytic strategies have been widely explored for polyester depolymerization, with both Brønsted bases and acids, and shown to catalyze chain scission under homogeneous conditions [24, 25, 26, 27, 28]. In this context, base‐mediated systems, specifically 4‐dimethylaminopyridine (DMAP) and imidazole, were examined. DMAP has previously been reported to promote efficient chain scission of PLA homopolymers via transesterification with an alcohol [29]. More generally, *N*‐heterocyclic bases are more active and even sometimes more selective for lactide versus ε‐caprolactone‐based transesterification reactions such as ring‐opening polymerization (ROP) [30, 31]. From this, we envisioned some selectivity for lactyl units in the corresponding degradation of L‐lactide/ε‐caprolactone chain‐shuttled copolymers via N‐heterocyclic base such as DMAP and imidazole‐catalyzed transesterification. On the other hand, ROP of lactones is well catalyzed by Brønsted acids [32, 33], whereas ROP of lactide needs harsher conditions with these compounds [34]. A selectivity for caprolactoyl segments may here be envisioned similarly in the course of the degradation of L‐lactide/ε‐caprolactone chain‐shuttled copolymers via transesterification. Subsequently, acid‐catalyzed degradation was investigated using p‐toluenesulfonic acid (pTSA) [35, 36] and diphenyl phosphate (DPP) [32, 37] as Brønsted acid catalysts known for their effectiveness in catalyzing transesterification reactions.

In this context, we report herein the degradation of L‐lactide/ε‐caprolactone chain‐shuttled copolymers using various enzymatic and organocatalytic strategies and discuss their relative performances and selectivities.

## 2 Results and Discussion

The starting statistical and chain‐shuttled copolymers, referred to as SS and CS, respectively, are presented in Table 1. Relevant information includes their composition and their molecular‐weight distribution. A typical ^1^H NMR spectrum is provided in the Figure S2 together with the DSC analysis.

**TABLE 1:** Designation, copolymer nature, composition (mol%), and number‐average molecular weight of the investigated samples.

Sample	Composition		M_*n*_	Dispersity, Đ
	PLLA	PCL	g/mol	
SS1	39%	61%	26,000	1.1
CS1	68%	32%	14,000	1.1
CS2	66%	34%	20,000	1.1
CS3	59%	41%	19,000	1.3

### 2.1 Enzymatic Degradation via Hydrolysis

To demonstrate the feasibility of degrading L‐lactide/ε‐caprolactone chain‐shuttled copolymers, initial experiments were conducted on the chain‐shuttled sample CS1, following conditions previously reported by Vert et al. [21] for the selective degradation of poly(ε‐caprolactone‐*co*‐L‐lactide) statistical copolymers, which were conducted in aqueous buffer (entry 1 in Table 2). ^1^H NMR analysis after degradation was found to be similar to that of the starting material. In addition, SEC analysis showed no clear shift in the chromatogram, corresponding just to 2% degradation yield. The same experimental conditions were conducted on the statistical sample SS1 (entry 2) to validate the experimental setup. SEC analysis (given in the Figure S3) showed a complete shift of the molecular‐weight distribution toward higher elution times, from the polymer region to the oligomer region. We also assessed a statistical copolymer with higher lactide content (see the section “Enzymatic degradation of statistical copolymer with high PLLA content” in the Supporting Information section) and observed that degradation was not observed in this case, following also the literature results [21]. These findings confirm the validity of the applied methodology and suggest that the limited degradation observed with the chain‐shuttled samples is related to their structural characteristics rather than the method itself. We further validated the reproducibility of the experiment by conducting duplicate (see the Duplicate Degradation Experiments section of the Supporting Information section).

**TABLE 2:** Enzyme‐catalyzed degradation of statistical (SS) and chain‐shuttled (CS) samples under varied conditions.^a^

Entry	Sample	(Co)solvent	Reaction time, days	Temperature, °C	Yield of degradation, wt%^b^
1	CS1	None	10	37	2
2	SS1	None	7	37	100
3	SS1	10% DMSO	7	37	100
4	SS1	10% DMF	7	37	100
5	CS1	10% DMSO	10	37	5
6	CS1	10% DMF	10	37	4
7	CS1	30% DMSO	10	37	8
8	CS1	30% DMSO	20	37	21
9	CS1	30% DMSO	10	60	22
10	CS1	100% DMSO	10	37	3
11	CS1	100% DMF	10	37	2

a Buffer as the main solvent (pH 7.6), 45 mg copolymer, and enzyme loading 1 mg.

b Determined by SEC analysis (see Experimental section for details).

The limited degradation of the chain‐shuttled copolymers is likely due to limited enzymatic accessibility to the copolymer. In the literature, numerous articles report the use of lipases in unconventional media, namely, in the presence of organic solvents [38]. Therefore, dimethyl sulfoxide (DMSO) and dimethylformamide (DMF) were added to the buffer as cosolvents to promote polymer chain mobility [23], thereby enhancing the enzymatic accessibility. To evaluate the tolerance of the enzyme toward the investigated cosolvents, degradation reactions were carried out first on the statistical copolymer SS1, which previously exhibited quantitative degradation in pure buffer. Reactions were first conducted in the presence of 10 vol% DMSO and 10 vol% DMF as cosolvents (entries 3 and 4, respectively). In both cases, degradation behavior comparable to that observed in buffer (entry 1) was observed, indicating quantitative degradation and demonstrating full enzyme tolerance toward these cosolvents at this concentration.

The use of cosolvent was subsequently extended to chain‐shuttled copolymer CS1. The effect of cosolvents was then examined under otherwise identical conditions using 10 vol% DMSO and 10 vol% DMF (entries 5 and 6, respectively). A slight increase in degradation was observed, with yields of 5% and 4%, respectively, suggesting a limited swelling effect of the cosolvents. To further enhance degradation, the co‐solvent loading was increased, and 30 vol% DMSO was investigated (entry 7). In this case, degradation increased to 8%, demonstrating an improvement relative to lower cosolvent loadings. Higher DMSO loadings were not explored as they led to its crystallization at room temperature upon mixing with the buffer.

Based on the improved degradation observed with 30 vol% DMSO as a cosolvent, this composition was retained while other reaction parameters were systematically varied. First, the reaction time was extended from 10 to 20 days (entry 8 vs. 7). This modification led to a clear improvement in degradation, with the yield increasing to 21%, indicating that the enzyme retained its activity over an extended reaction period. The effect of temperature was then investigated by increasing the reaction temperature to 60 °C, a temperature at which the enzyme is reported to remain active [39]. Under these conditions, the degradation yield increased from 8 (entry 7, 37 °C) to 22% (entry 9, 60 °C). This result was evidenced by the appearance of chain end signals in the ^1^H NMR spectrum after degradation, characteristic chain‐end signals of PLLA (quartet at 4.35 ppm) and PCL (triplet at 3.65 ppm), as well as by an increased area of the oligomeric fraction in the SEC chromatogram (Figures 2 and 3, respectively).

**FIGURE 2:**
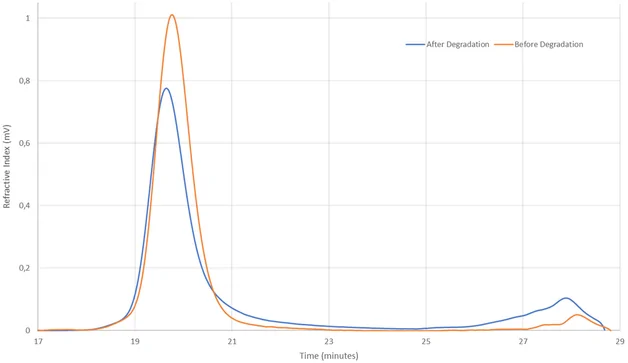
SEC chromatograms of CS1 before and after degradation (entry 9).

**FIGURE 3:**
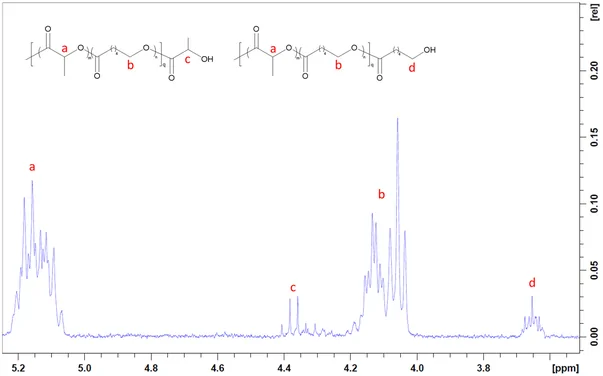
Expanded ^1^H NMR spectrum of degraded CS1 – entry 9 (300 MHz, CDCl_3_) in the 3.6–5.2 ppm region.

Although improvement in the degradation was achieved, reaching 21%–22%, this value remains insufficient for both recycling and analytical purposes. As the limiting factor appears to be the enzymatic accessibility to the polymer, we next moved away from buffer‐based systems, which result in heterogeneous reaction conditions. Instead, degradation experiments were conducted in pure organic solvents, including DMSO and DMF, to establish homogeneous conditions and improve polymer–enzyme contact (entries 10 and 11, respectively). However, in both cases, only negligible degradation was observed. This outcome is understandable as enzymes may retain their activity in pure organic solvents; however, solvent‐induced conformational changes can significantly alter enzymatic activity and selectivity depending on the reaction medium [40, 41, 42].

### 2.2 Organocatalyzed Chain Scission via Transesterification

Base‐catalyzed degradation was carried out on the chain‐shuttled copolymer CS1 using basic catalysts, namely, DMAP and imidazole (Figure 4, entries 12 and 13, respectively), under conditions similar to those reported in the literature for the degradation of PLA homopolymers using DMAP [8]. Ethanol was used as nucleophile, and the reaction therefore proceeded via transesterification. No precipitation occurred during the workup, indicating complete conversion of the polymer into soluble degradation species. The degradation products were thus recovered directly from the homogeneous solution.

**FIGURE 4:**
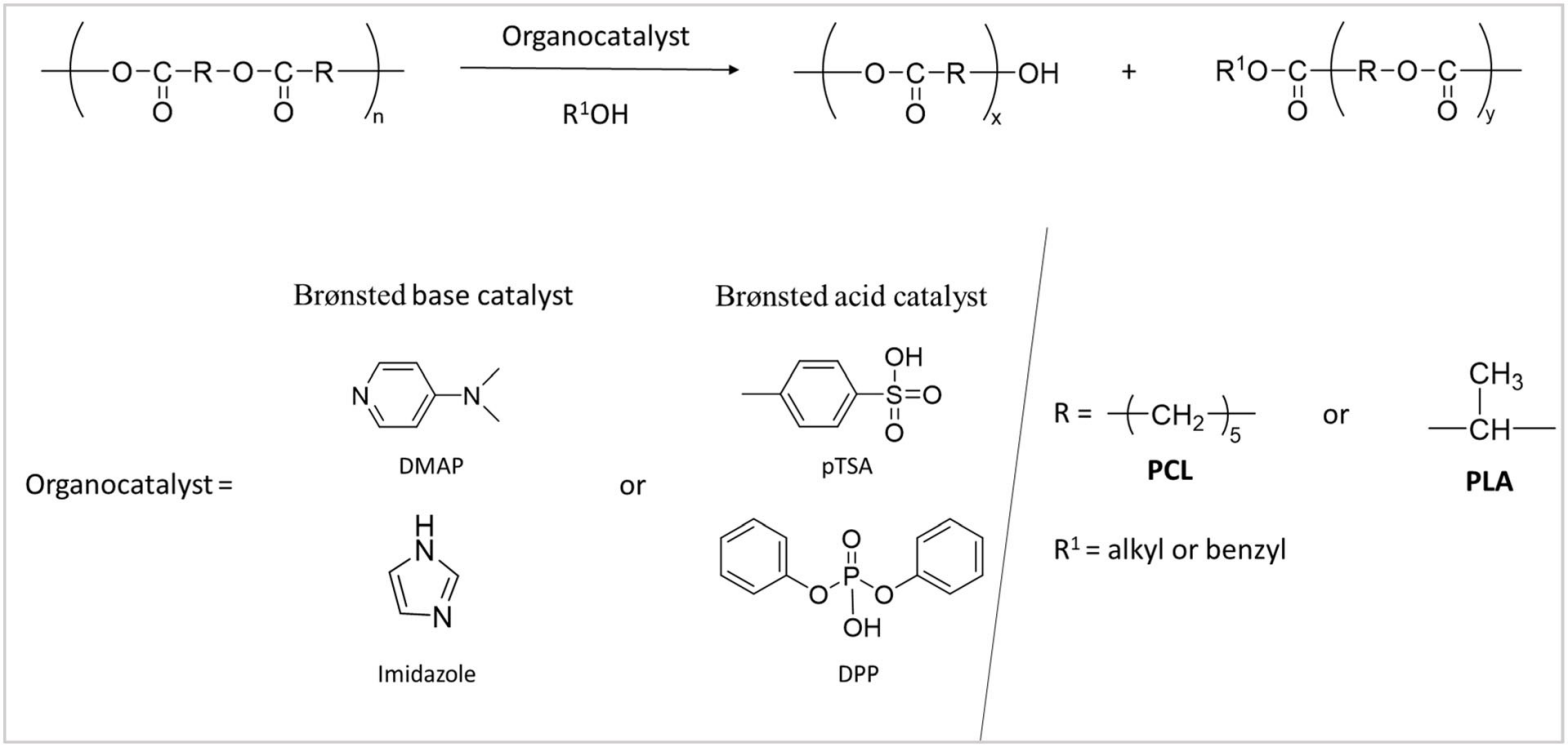
Transesterification of L‐lactide/ε‐caprolactone chain‐shuttled copolymers using organocatalysts.

In both cases, ^1^H NMR analysis of the soluble fraction revealed a significant PLLA chain‐end signal at approximately 4.35 ppm, whereas the PCL chain‐end signal at 3.65 ppm was not detected (Figure 5 – Table 3, entry 12). This indicates the selective degradation of the lactyl units. SEC analysis of the degradation products revealed a shift of the elution peaks toward higher retention times (Figure 6 – Table 3, entry 12). This is representative of oligomers with a broad molecular‐weight distribution. Overall, this methodology enables complete conversion of chain‐shuttled copolymers into ethanol‐soluble degradation products via selective transesterification of lactyl units, albeit with a broad molecular‐weight distribution. As such, it follows the selectivity observed for *N*‐heterocyclic base‐catalyzed ROPs, as discussed in the Introduction section, where lactide is polymerized selectively and quantitatively from a lactide/ε‐caprolactone mixture [30]. Finally, it should be noted that blank experiments conducted in similar experimental conditions (found in the Supporting Information section) showed no significant degradation.

**FIGURE 5:**
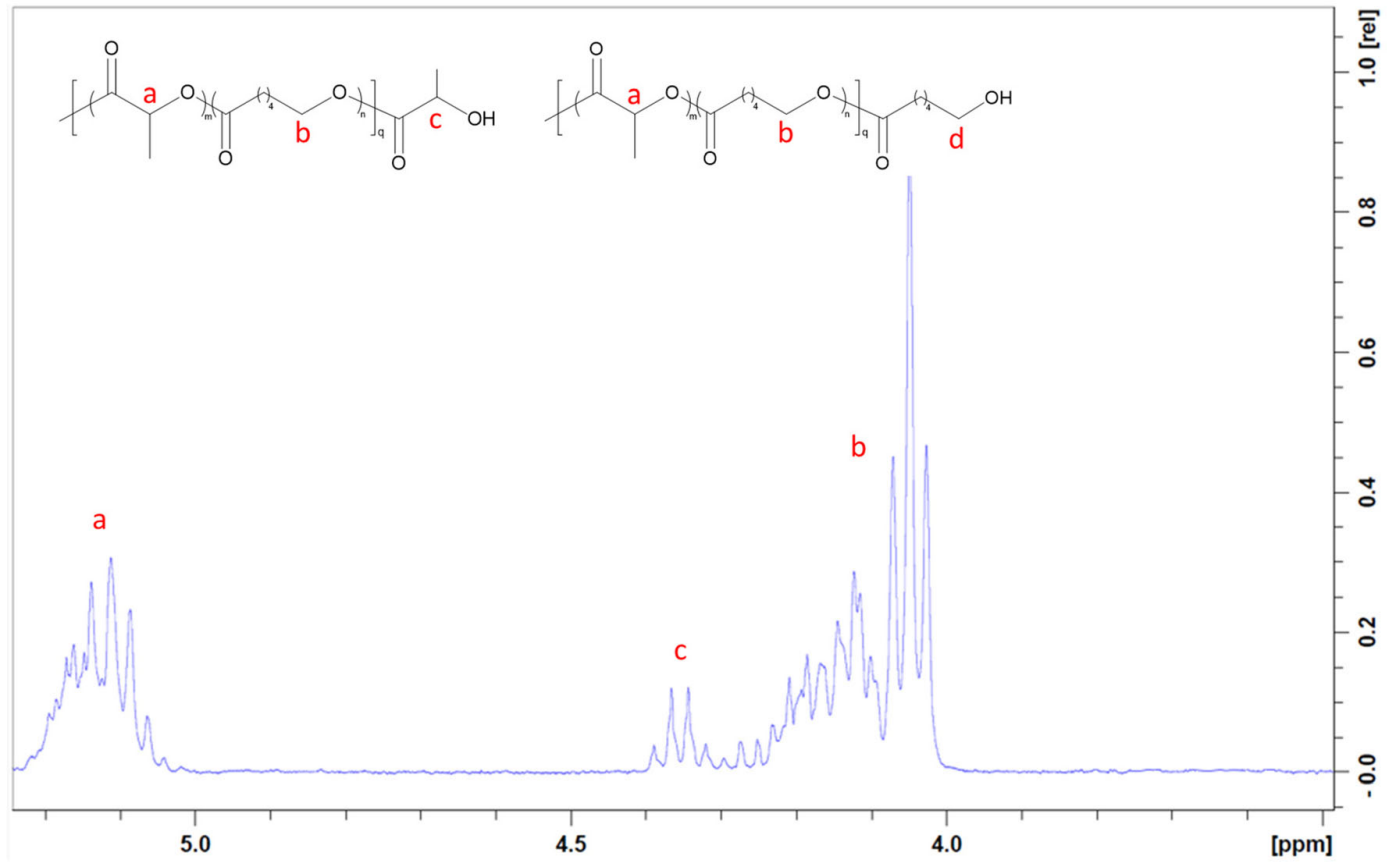
Expanded ^1^H NMR spectrum of degraded CS2 – entry 12 (300 MHz, CDCl_3_) in the 3.6–5.2 ppm region.

**TABLE 3:** Organocatalytic chain scission of L‐lactide/ε‐caprolactone chain‐shuttled copolymers.

Entry	Sample	Catalyst nature	Catalyst	Nucleophile	DD_PLLA_ a	DD_PCL_ a	Selectivity to PCL, mol%^a^
12	CS2	Brønsted base^b^	DMAP	Ethanol	10.9	0	0
13	CS2		Imidazole	Ethanol	9	0	0
14	CS2	Brønsted acid^c^	pTSA	Ethanol	4.3	5.7	57
15	CS3		pTSA	Benzyl alcohol	5	9.7	66
16	CS2		pTSA	1‐Octanol	2.7	7.3	73
17	CS3		DPP	1‐Octanol	1.2	8.8	88

a Calculation method given below in details.

b Degradation conditions (2 days, 38 °C, ethanol (0.5 mol eq to ester groups), basic catalyst (0.2 mol eq to ester groups), DCM as solvent).

c Degradation conditions (6 hours, 60 °C, nucleophile (1 mol eq to ester groups), acidic catalyst (1/40 mol eq to monomeric unit), toluene as solvent).

**FIGURE 6:**
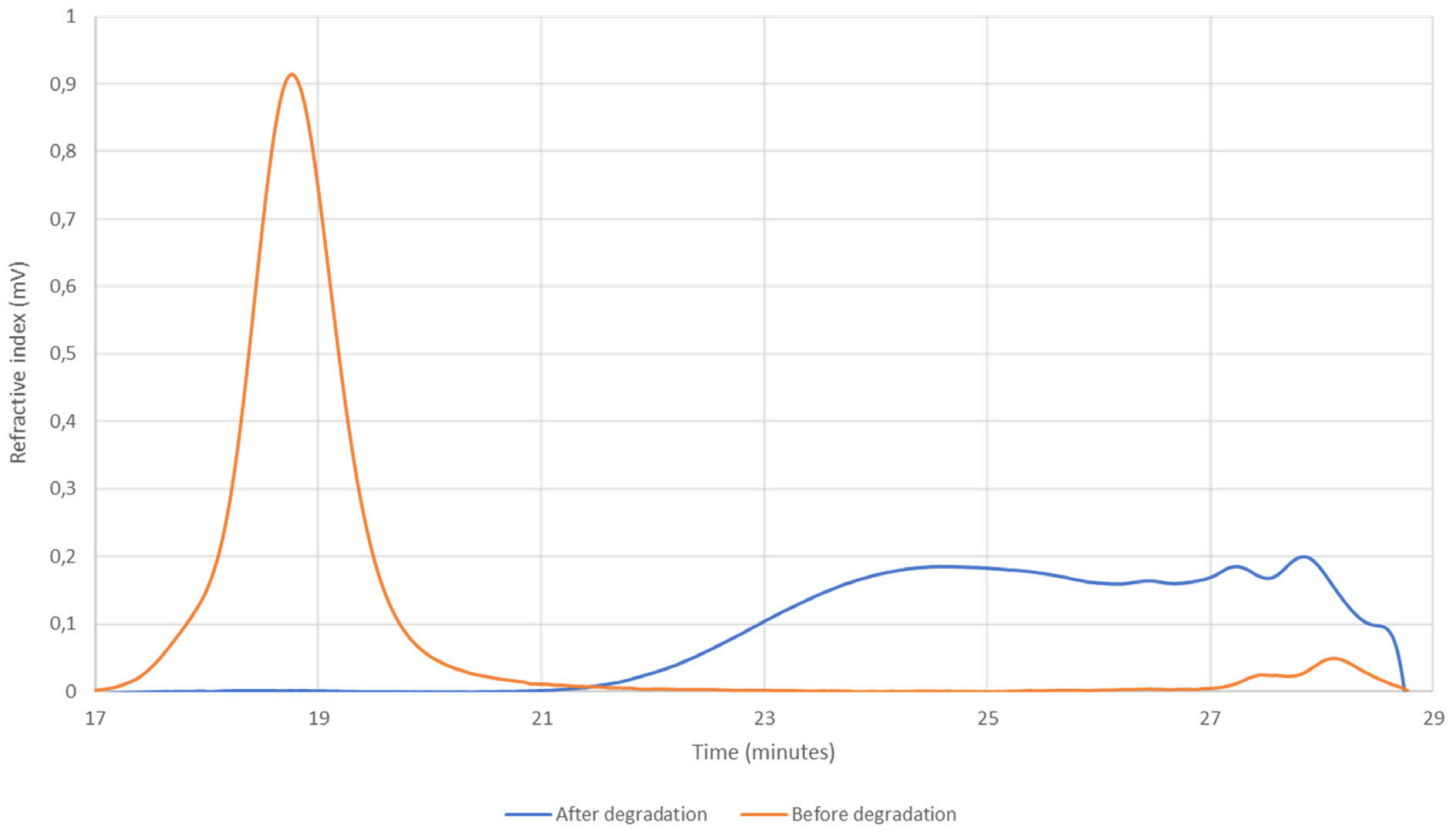
SEC chromatograms of CS2 before and after degradation (entry 12).

Brønsted acid‐catalyzed degradation was subsequently investigated. pTSA, known as an effective transesterification catalyst [35, 36], was employed as the acid catalyst for the degradation of L‐lactide/ε‐caprolactone chain‐shuttled copolymers by transesterification. Different alcohols (entries 14–16 in Table 3) were used as nucleophiles for this purpose. From the SEC curves provided in Figures S5–S7, complete conversion of the starting material was achieved under all conditions, and no precipitation was observed during the workup. Regardless of the nucleophile employed, ^1^H NMR analysis revealed the presence of signal characteristics to both PLLA and PCL chain ends (Figure 7), indicating that pTSA degrades both polymeric components. Importantly, ^1^H NMR analysis revealed that the relative extent of degradation of each polymer segment varies depending on the alcohol employed, indicating a degree of selectivity in the transesterification process. This prompted us to assess the degradation selectivity. Blank experiments conducted in similar experimental conditions (found in the Supporting Information section) showed no significant degradation.

**FIGURE 7:**
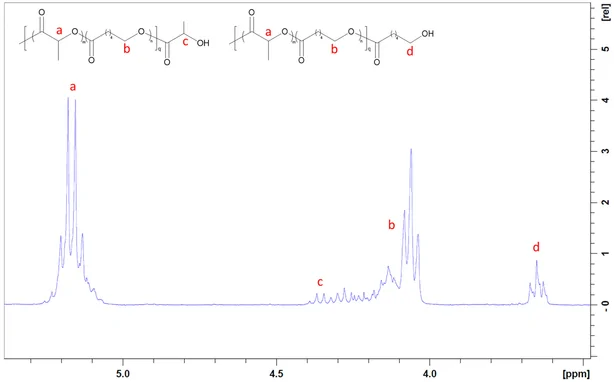
Expanded ^1^H NMR spectrum of degraded CS2 – entry 16 (300 MHz, CDCl_3_) in the 3.6–5.2 ppm region.

The degradation selectivity was quantitatively determined using an ^1^H NMR‐based approach and is expressed in mol%. The selectivity of the reaction for caprolactoyl transesterification can be determined as follows:

(1) $$S_{\mathrm{PCL}}=\frac{\frac{1}{2}I_{\mathrm{PCL}\text{ chain}\;\mathrm{end}}}{I_{\mathrm{PLLA}\text{ chain}\;\mathrm{end}}+\frac{1}{2}I_{\mathrm{PCL}\text{ chain}\;\mathrm{end}}}\times 100$$

The degree of degradation (DD) of lactyl units can also be determined from the ^1^H NMR analysis:

(2) $$DD_{\mathrm{PLLA}}=\frac{I_{\mathrm{PLLA}\text{ chain}\;\mathrm{end}}}{I_{\mathrm{PLLA}\text{ chain}}+I_{\mathrm{PLLA}\text{ chain}\;\mathrm{end}}}\times 100$$

Then, we can determine the DD of caprolactoyl units using Equation (4) derived from Equation (3).

(3) $$S_{\mathrm{PCL}}=\frac{DD_{\mathrm{PCL}}}{DD_{\mathrm{PCL}}+DD_{\mathrm{PLLA}}}\times 100$$

(4) $$DD_{\mathrm{PCL}}=\frac{S_{\mathrm{PCL}}\times DD_{\mathrm{PLLA}}}{1-S_{\mathrm{PCL}}}\times 100$$

The DD is a key parameter that represents the extent of degradation relative to each monomeric unit determined on a molar basis. In these equations, the *I*_PLLA_ chain is the integral value of the lactyl *‐CH‐* signal at 5.2 ppm, while the *I*_PLLA_ chain end and the *I*_PCL_ chain end represent the integral values of the lactyl *–CH–* chain end resonance at 4.35 ppm and the caprolactoyl *CH_2_–O* chain end resonance at 3.65 ppm, respectively (see Figure 7).

The selectivity of the reaction for caprolactoyl transesterification is given in Table 3. All the alcohols tested with pTSA exhibited higher selectivity toward PCL degradation. Among them, bulkier alcohols such as benzyl alcohol (66%, entry 15) and especially 1‐octanol (73%, entry 16) led to the highest selectivity for transesterification with caprolactoyl units. This size effect may be explained by the steric hindrance around PLLA ester bonds due to its methyl substituents, which makes them less accessible to bulkier alcohols. As a result, bulkier alcohol molecules are less likely to react with lactyl units and more likely to target the more accessible caprolactoyl units, thus improving selectivity. In addition, another Brønsted acid catalyst, DPP, was investigated under the same conditions using 1‐octanol as a nucleophile, which previously showed the highest selectivity with pTSA. Under these conditions, DPP also exhibited high selectivity toward caprolactoyl unit degradation, reaching 88% (entry 17), exceeding that obtained with pTSA.

We further validated the reproducibility of the experiment by conducting duplicate experiments (see the Duplicate Degradation Experiments section of the Supporting Information).

### 2.3 Brönsted Acid Catalyzed Transesterification of the Chain‐Shuttled Copolymers: Mechanistic and Microstructural Insights

The selectivity of the Brönsted acid‐catalyzed reaction is of interest for our materials, as it allows the degradation of preferentially the soft, poly(L‐lactide‐*co*‐ε‐caprolactone) blocks of the copolymer. We thus decided to perform DFT studies on model molecules to get mechanistic insights. PCL and polylactic acid (PLA) fragments were represented by n‐propyl propanoate and iso‐propyl isopropanoate, respectively. We considered a dual activation reported to occur preferentially in our previous work on pTSA‐catalyzed PET synthesis via transesterification [36]. This involves the acidic activation of the carbonyl plus the basic activation of the alcohol, as represented in Scheme 1.

**SCHEME 1:**
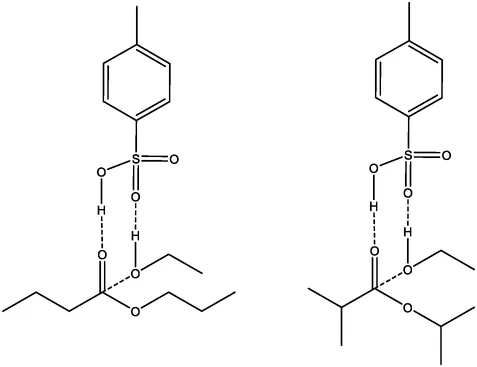
Dual activation mechanism considered in this study for pTSA catalyzed transesterifications.

For the pTSA‐catalyzed PCL model case, the reaction begins with a doubly hydrogen–bonded complex. One hydrogen bond is formed between a sulfonyl oxygen atom of PTSA and the alcohol hydroxyl group, while a second hydrogen bond connects the acidic hydroxyl group of PTSA to the carbonyl oxygen of the ester substrate. The first step involves a concerted proton‐exchange/nucleophilic‐addition process, in which the ethoxy moiety attacks the carbonyl carbon while the hydrogen‐bond network is reorganized. This transformation proceeds through **TS1** (Figure 8) with an activation free energy of 14.6 kcal mol^−1^, yielding a tetrahedral intermediate. This step is the rate‐determining step for this mechanism. The energy profile is provided in Figure 9.

**FIGURE 8:**
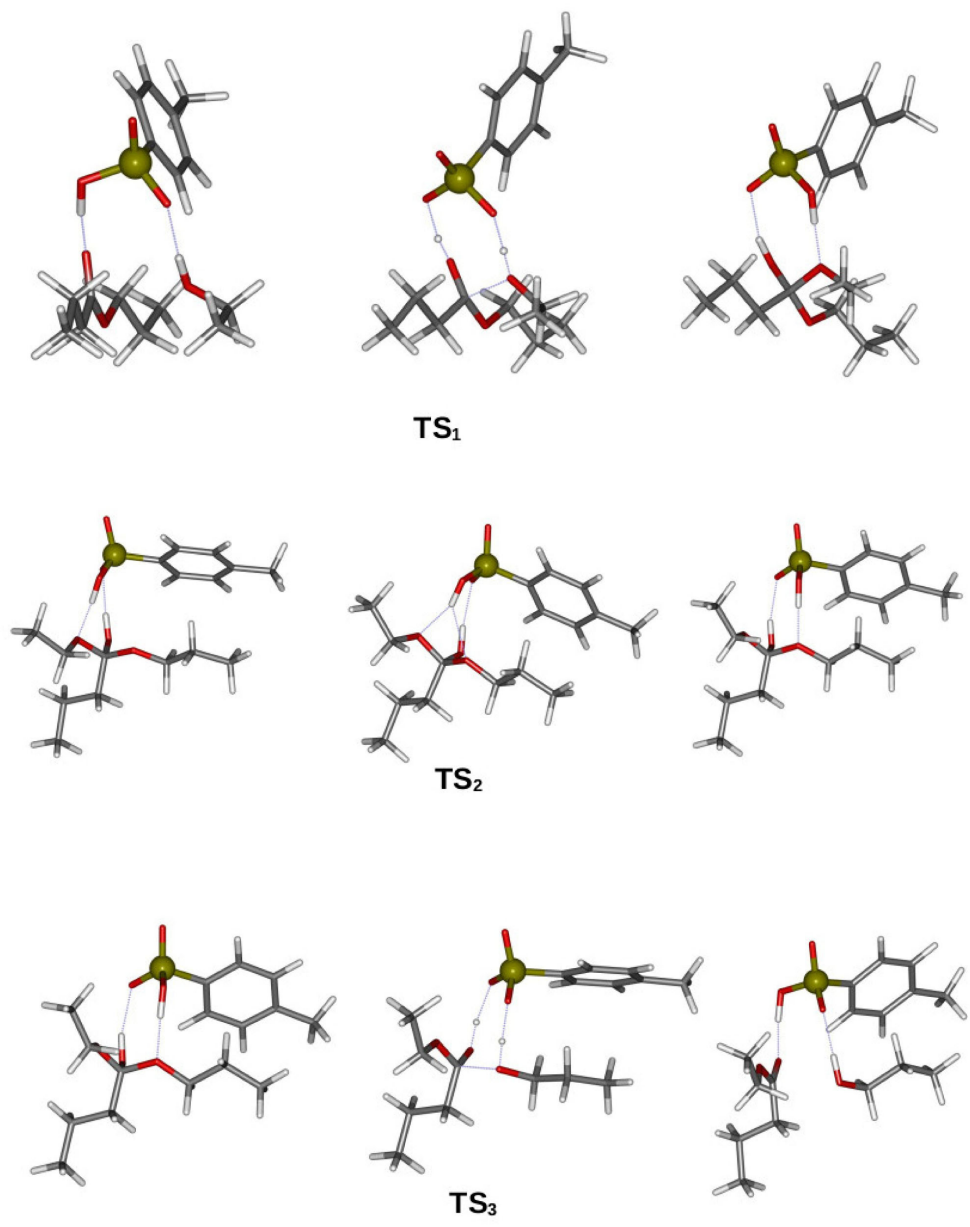
Optimized geometry structures of the species involved in the mechanism for the pTSA catalyzed PCL reaction Color code: C gray, O red, S yellow, and H white.

**FIGURE 9:**
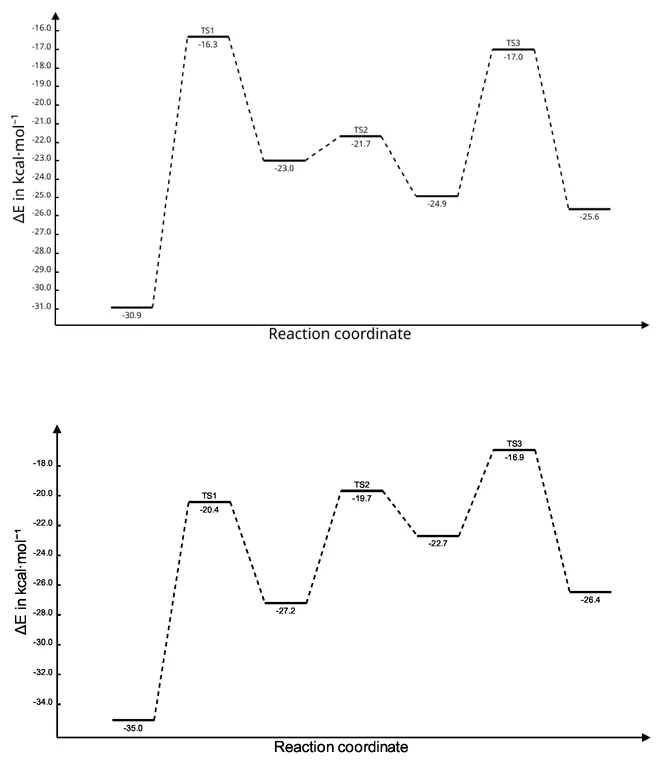
Energy profiles for the PTSA catalyzed caprolactoyl (top) and lactyl (bottom) transesterifications.

The reaction then proceeds by migration of the hydrogen bond from the ethyl ester oxygen to the propyl ester oxygen via **TS2** (Figure 8), a process associated with a negligible barrier. In the final step, simultaneous proton transfer and C─O bond cleavage take place. The acidic hydroxyl group of PTSA protonates the propyl ester oxygen, while the protonated carbonyl‐derived hydroxyl group transfers its proton back to the catalyst, leading to product formation and catalyst regeneration. This step proceeds through **TS3** (Figure 8) and requires an activation free energy of 14 kcal mol^−1^ relative to the reactant adduct. The same general mechanistic features are obtained for the PLA model (optimized structure is provided in the Figure S11), although the calculated barrier heights differ (see Figure 9, bottom), indicating a substrate‐dependent modulation of the reaction energetics. For this reaction, the last step (**TS3**) is the rate‐determining step. Comparing the highest energy barriers for both reactions, the reaction involving PCL seems more favorable, as observed experimentally.

The use of 1‐octanol as the degrading agent resulted in high selectivity toward caprolactoyl unit degradation, reaching 73% in combination with pTSA for CS2 and 88% with DPP for CS3 (entries 16 and 17, respectively), indicating preferential degradation of the soft blocks. On this basis, the molecular weight of the longest PLLA‐rich hard blocks could be roughly estimated by assuming that transesterification of lactyl units occurs predominantly within the soft segments, i.e., the statistical poly(ε‐caprolactone‐*co*‐L‐lactide) copolymer. This assumption is supported by the higher accessibility of the soft domains, as reflected by the preferential degradation of caprolactoyl units. As such, the contribution of low‐molecular‐weight oligomers, attributed mainly to degraded soft segments, was removed from the SEC traces by peak deconvolution using the freeware Fityk software, and we considered only the highest part of the distribution as an indication of the molecular weight of the longest PLLA blocks (see Figures S9 and S10). The resulting distributions were then used to recalculate a number‐average molecular weight of the longest hard blocks (M_*n*_^LHB^). Accordingly, M_*n*_^LHB^ values of 5400 and 3000 g·mol^−1^ were obtained for samples CS2 and CS3 under pTSA/1‐octanol (entry 16, 73% selectivity) and DPP/1‐octanol (entry 17, 88% selectivity) conditions, respectively.

## 3 Conclusion

In this work, the degradation of L‐lactide/ε‐caprolactone chain‐shuttled multiblock copolymers was investigated with the idea to find selectivity, especially for caprolactoyl units. Enzymatic degradation using free lipase from *P. cepacia* showed limited efficiency in this context, probably due to restricted accessibility induced by the complex multiblock architecture. Homogeneous organocatalytic approaches were then explored. Base‐catalyzed degradation using Brønsted bases enabled efficient chain scission via transesterification and exhibited selectivity toward lactyl unit degradation. In contrast, Brønsted acid catalysis enabled the selective degradation of the caprolactoyl units via transesterification. The selectivity observed was corroborated by DFT for pTSA as a case study via a dual‐activation mechanism. Overall, this study demonstrates that the proper choice of organocatalysts can enable to choose the selectivity of the transesterification of L‐lactide/ε‐caprolactone chain shuttled multiblock copolymers with alcohols and can serve as a valuable approach for the structural investigation of complex materials prepared via chain‐shuttling polymerization. Finally, the process may be applied in the future for recycling of the polylactide part as lactide is produced from PLA oligomers.

## Funding

This work was supported by the Agence Nationale de la Recherche (Grant ANR‐21‐CE06‐0024).

## Conflicts of Interest

The authors declare no conflicts of interest.

## Supporting information

Supplementary Material

## Data Availability

The data that support the findings of this study are available on request from the corresponding author. The data are not publicly available due to privacy or ethical restrictions.
