# Supplementary material for: Selective Chain Scission of L‐Lactide/ε‐Caprolactone Chain Shuttled Copolymers
Source: ChemSusChem. 2026 Oct 30;19(21):e71174. doi: 10.1002/cssc.71174 (PMC13722158; doi:10.1002/cssc.71174)
Supplement: Supplementary file 1 [file CSSC-19-e71174-s001.pdf]

Supporting Information

Selective Chain Scission of L-lactide / ε-caprolactone Chain Shuttled Copolymers

A. Noon,[a] L. Fichaux,[a] X. Mosca,[a] P. Lowy,[a] T. Bousquet,[a] L. Pelinski,[a] F. Bonnet,[b] Joao P. Prates Ramalho,[c] A. Favrelle-Huret,[a] P. Zinck*[b]

[a] UCCS – Unité de Catalyse et Chimie du Solide (UMR 8181), Univ. Lille, CNRS, Centrale Lille, Univ. Artois, 59650 Villeneuve d'Ascq, France

Email : philippe.zinck@univ-lille.fr

[b] UMET - Unité Matériaux et Transformations (UMR 8207), Univ. Lille, CNRS, INRAE, Centrale Lille, 59000 Lille, France

[c] Chemistry Department, LAQV-REQUIMTE and Hercules Centre, Sciences and Technology School, University of Evora, ´ Rua Romao ˜ Ramalho 59, 7000-671 Evora, Portugal

Experimental Part

Materials

Free lipase from *Pseudomonas cepacia* (activity >30 U·mg^{-1}) was purchased from Sigma-Aldrich. Sodium dihydrogen phosphate (98%) was obtained from Fisher Scientific, and disodium hydrogen phosphate (>99%) from VWR Chemicals. Dimethyl sulfoxide (DMSO, HPLC grade) and dimethylformamide (DMF, 99%) were purchased from Alfa Aesar. Toluene was purified using an MBraun solvent purification system. Dichloromethane (DCM, 98%) and absolute ethanol were obtained from VWR Chemicals. 4-Dimethylaminopyridine (DMAP, 99%) was purchased from Acros Organics. Imidazole (pure) was obtained from Sigma-Aldrich. *p*-Toluenesulfonic acid (pTSA, 97%) was obtained from Alfa Aesar. 1-octanol (anhydrous, >99%) and benzyl alcohol (anhydrous, 99%) were purchased from Sigma-Aldrich. All chemicals were used as received unless otherwise stated. L-lacide / ε-caprolactone chain shuttled copolymers were synthesized as described in our previous work.[1] The DSC analysis of the chain shuttled copolymer is provided below.

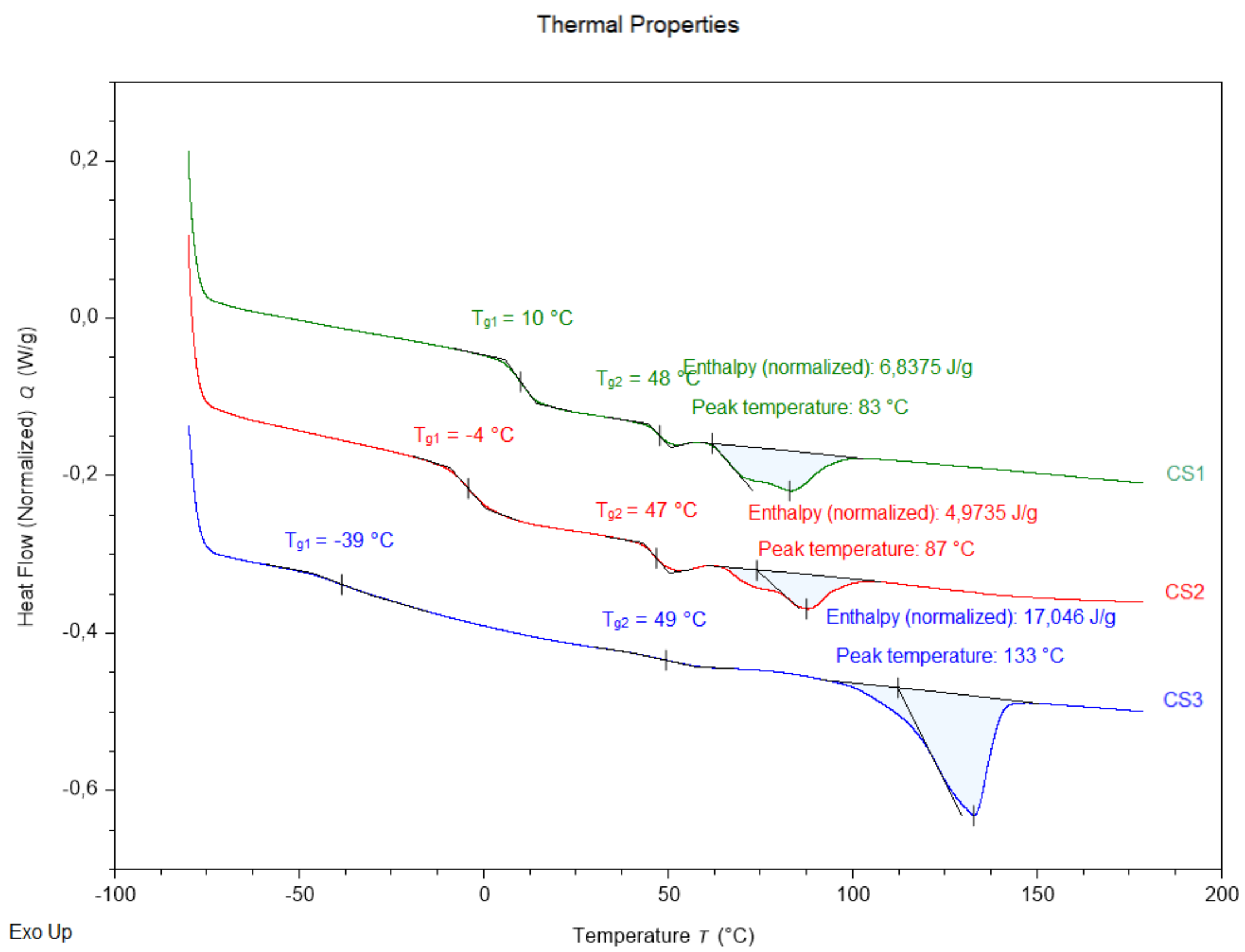

[1] X. Mosca *et al.*, "Tuning the thermal properties of l-lactide/ϵ-caprolactone chain shuttled copolymers via catalyst selection," *Polym. Chem.*, vol. 15, no. 39, Sep. 2024, doi: 10.1039/d4py00671b

Procedures

Enzymatic Degradation

Polymeric films were prepared by solvent casting. The copolymer (45 mg) was dissolved in dichloromethane (DCM, 1 mL) under magnetic stirring until complete dissolution. The solution was poured into a Teflon Petri dish (Ø 40 mm, depth 25 mm) and the solvent was allowed to evaporate under a fume hood for 24 h to obtain uniform films.

The films were placed in a 50 mL round bottom flask together with free lipase from *Pseudomonas cepacia* (activity >30 $U \cdot mg^{-1}$; typically 1 mg) and 5 mL of solvent (buffer/co-solvent mixtures). The reaction mixture was stirred at 900 rpm in an oil bath at 37–60 °C for 7 days (statistical copolymers) or 10–20 days (chain shuttled copolymers).

After completion of the reaction, solvents were removed under reduced pressure using a rotary evaporator. Buffer/DMF mixtures were removed directly, whereas DMSO-containing systems were further dried under high vacuum due to the high boiling point of DMSO (189 °C). The residue was dissolved in DCM (5 mL) to ensure homogeneity, and the solvent was evaporated under a fume hood for 24 h. The recovered materials were analyzed by ^{1}H NMR spectroscopy and size exclusion chromatography (SEC).

Preparation of Buffer solution

A phosphate buffer solution (target concentration 0.05 $mol \cdot L^{-1}$, pH 7.6) was prepared using sodium dihydrogen phosphate and disodium hydrogen phosphate as the conjugate acid–base pair (pKa = 7.2). The required ratio of the two salts was calculated using the Henderson–Hasselbalch equation. Sodium dihydrogen phosphate (0.17 g) and disodium hydrogen phosphate (1.28 g) were dissolved in 200 mL of deionized water to obtain the buffer solution.

Base Catalyzed Degradation

In a 50 mL reactor, the copolymer (75 mg) and the basic catalyst (DMAP or imidazole; 0.2 equiv. relative to ester groups) were dissolved in dichloromethane (DCM, 0.375 mL). Ethanol (0.5 equiv. relative to ester groups) was then added. The reactor was sealed, and the reaction mixture was stirred at 400 rpm in an oil bath at 38 °C for 2 days. Upon completion of the reaction, the mixture was added dropwise to cold ethanol where no precipitation was observed.

The filtrate was concentrated under reduced pressure to remove ethanol. The remaining solid residue was collected and characterized by ^{1}H NMR and SEC.

Acid Catalyzed Degradation

In a 50 mL reactor, the copolymer (75 mg) and p-toluenesulfonic acid (pTSA; 1/40 equiv. relative to monomer units) were dissolved in toluene to achieve a 1 mol·L^{-1} concentration of monomeric units. The selected degrading agent (alcohol; 1 equiv. relative to ester groups) was then added, and the reaction mixture was stirred at 60 °C for 6 h in an oil bath. Upon completion of the reaction, the mixture was added to ethanol where no precipitation was observed. The filtrate was concentrated under reduced pressure to remove ethanol. The recovered materials were characterized by ^{1}H NMR and SEC.

Characterization Techniques

Proton Nuclear Magnetic Resonance (Proton NMR)

The ^{1}H NMR spectra were recorded using a Bruker ADVANCE III HD 300 spectrometer operating at 300.13 MHz (7.05 Tesla) in deuterated chloroform ($CDCl_3$) at 298 K. The NMR sample was prepared by dissolving approximately 5 mg of the sample in 0.6 mL of $CDCl_3$. Data acquisition and analysis were performed using Bruker Topspin 4.3 software.

Size Exclusion Chromatography (SEC)

SEC analysis was performed using tetrahydrofuran (THF) as the eluent at 40°C with a flow rate of 1 mL/min. The system consisted of a Waters SIS HPLC pump, a Waters 2414 refractometer, and Waters Styragel columns HR3 and HR4. Calibration was conducted using polystyrene standards. The refractometer was used to detect the refractive index of the samples at various elution times. For sample preparation, 10 mg of the sample was dissolved in a mixture of 5 mL THF and toluene (1 mL toluene/1 L THF) to achieve a concentration of 2 g/L.

Calculation Methods

Calculation of the yield of degradation:

SEC can be utilized to determine the specific yield of degradation. This is achieved by calculating the surface area under the oligomeric / polymeric peaks before and after degradation (see e.g., Figure S3). The surface areas of each peak are determined using the trapezoidal rule. For this, the SEC data was reprocessed in an Excel spreadsheet, allowing us to calculate the surface areas under the peaks of interest. The formula for the trapezoidal rule is presented below, along with a graphical representation of the rule on a curve (Figure).

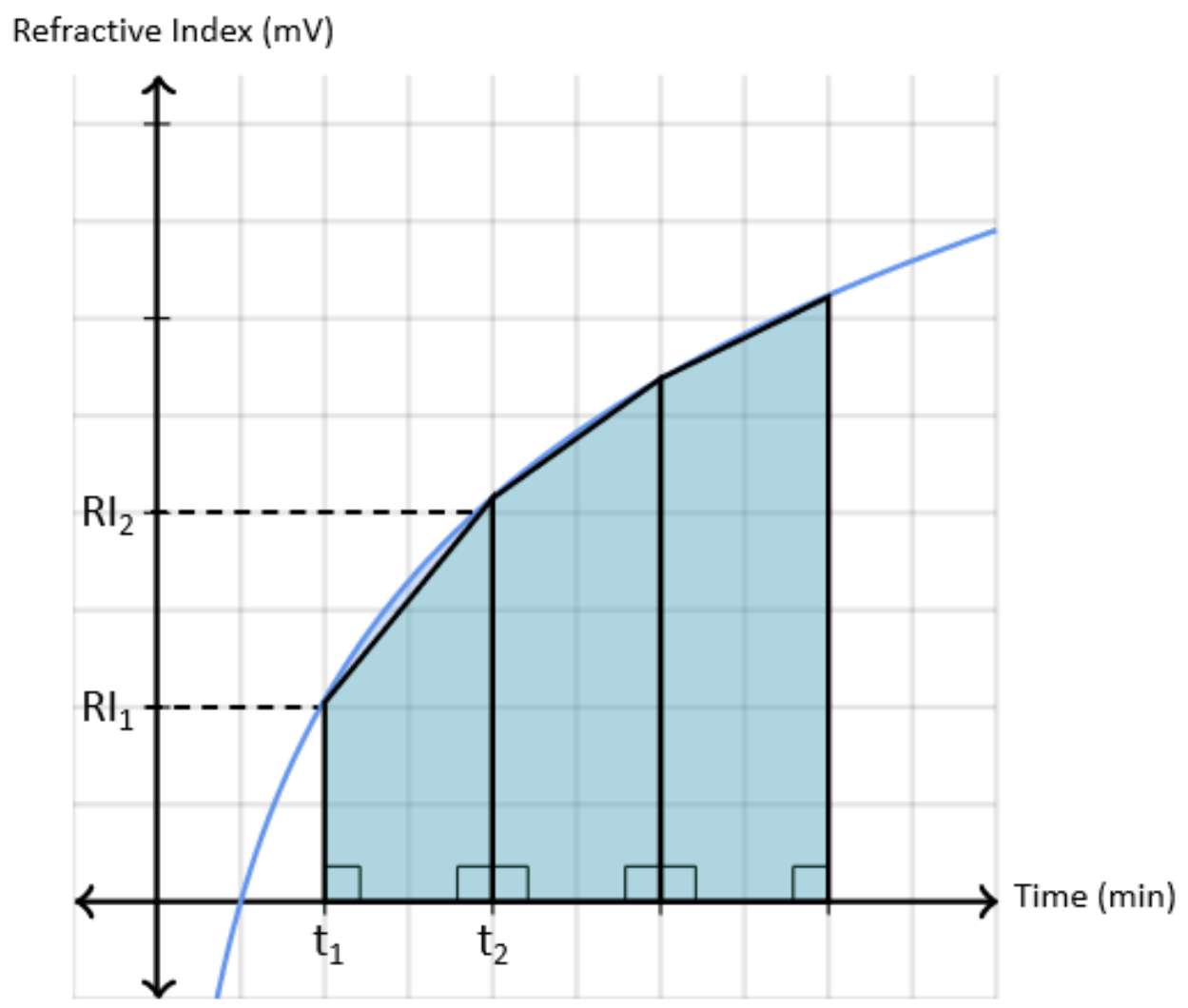

Figure S1. Representation of trapezoidal rule.

The areas under the peaks of interest, relative to oligomeric and polymeric peaks in the curve after degradation, are calculated using the trapezoidal rule as shown in **Equation S1** below.

$$S = (t_2 - t_1)\,.\left(\frac{RI_1 + RI_2}{2}\right) \qquad (S1)$$

Where *t* represents retention time in minutes, *RI* represents refractive index in mV, and *S* represents the surface area under the peak. The values determined by the trapezoidal rule are then utilized to determine the specific yield of degradation on a weight basis (wt%). The degradation yield was determined from the SEC chromatogram after degradation as the ratio of the oligomer peak area, corresponding to degradation products, to the total peak area (oligomer + polymer), representing all species present in the system. The yield formula is represented in **Equation S2** below.

$$Yield = \frac{S_{oligomer}}{S_{oligomer} + S_{polymer}} * 100 \qquad (S2)$$

For greater precision, this rule is applied to the curve before degradation, and then the two values are subtracted, because usually a small peak relative to oligomers exist before degradation.

Computational details

In our computational studies, a simplified model system was employed using methanol as the degrading agent and PTSA and DMAP as organocatalysts. Poly(ε-caprolactone) (PCL) and polylactic acid (PLA) fragments were represented by n-propyl propanoate and iso-propyl isopropanoate, respectively.

All quantum chemical calculations were carried out using the ORCA quantum chemistry package.[2] Geometry optimizations of all stationary points, including reactants, intermediates, products, and transition states, were performed within the framework of density functional theory (DFT) employing the range-separated hybrid functional wB97X-D3 in combination with the def2-SVP basis set. The selected functional includes empirical dispersion corrections and has demonstrated reliable performance for the description of thermochemistry, reaction energetics, and noncovalent interactions, particularly in organocatalytic systems where dispersive and hydrogen-bonding interactions play an important role.

Solvent effects were incorporated through the conductor-like polarizable continuum model (CPCM) using the parameters for dichloromethane (DCM) and toluene, depending on the system under investigation. All optimized geometries were characterized by harmonic vibrational frequency calculations. Minima on the potential energy surface were confirmed by the absence of imaginary frequencies, whereas transition-state structures exhibited a single imaginary frequency associated with the corresponding reaction coordinate. Intrinsic reaction coordinate (IRC) calculations were additionally performed to verify the proper connection between transition states and reactants and products. Molecular geometries and computed energetic data were analyzed and visualized using ChemCraft.[3]

[2] Neese, F. , Software update: the ORCA program system, version 6.0 WIRES Comput. Molec. Sci. 2025 15(1), e70019doi.org/10.1002/wcms.70019
[3] Zhurko G. A. Chemcraft - graphical program for visualization of quantum chemistry computations. Ivanovo, Russia, 2005. https://chemcraftprog.com

^{1}H NMR analysis of the chain shuttled copolymer CS1

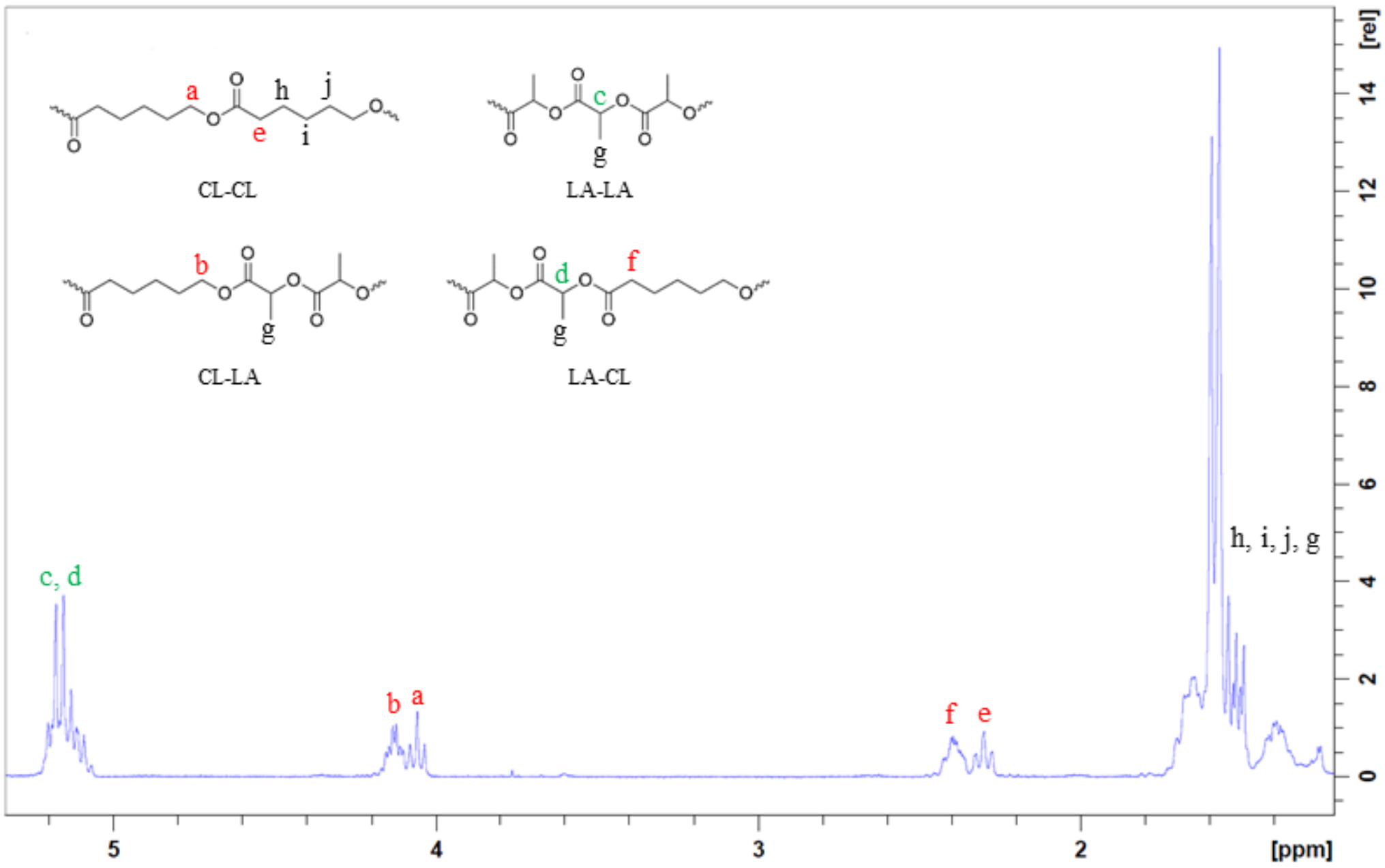

Figure S2. ^{1}H NMR spectrum of CS1 (300 MHz, $CDCl_3$).

SEC Chromatograms

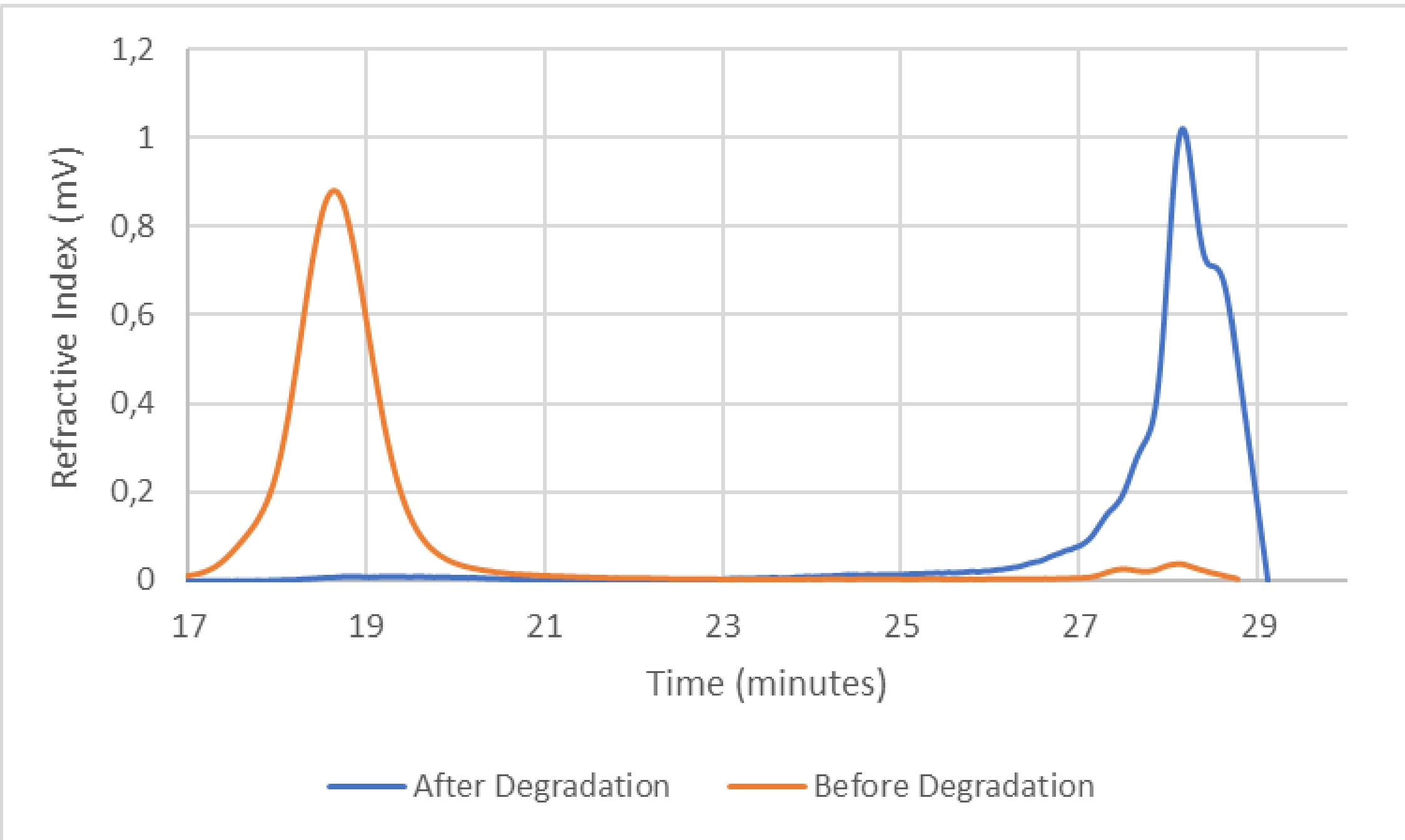

Figure S3. SEC chromatograms of SS1 before and after degradation (entry 2).

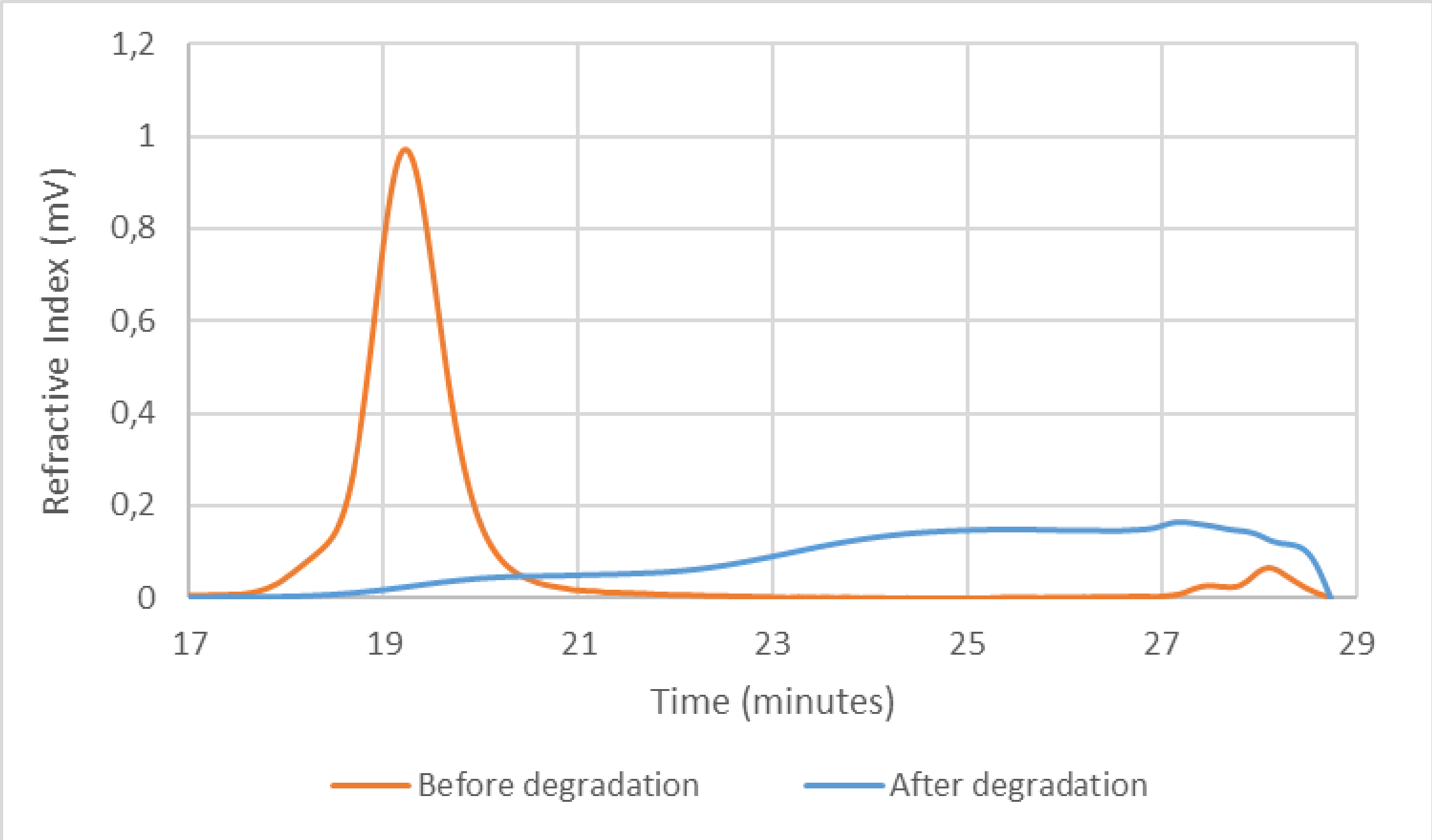

Figure S4. SEC chromatograms of CS2 before and after degradation (entry 13).

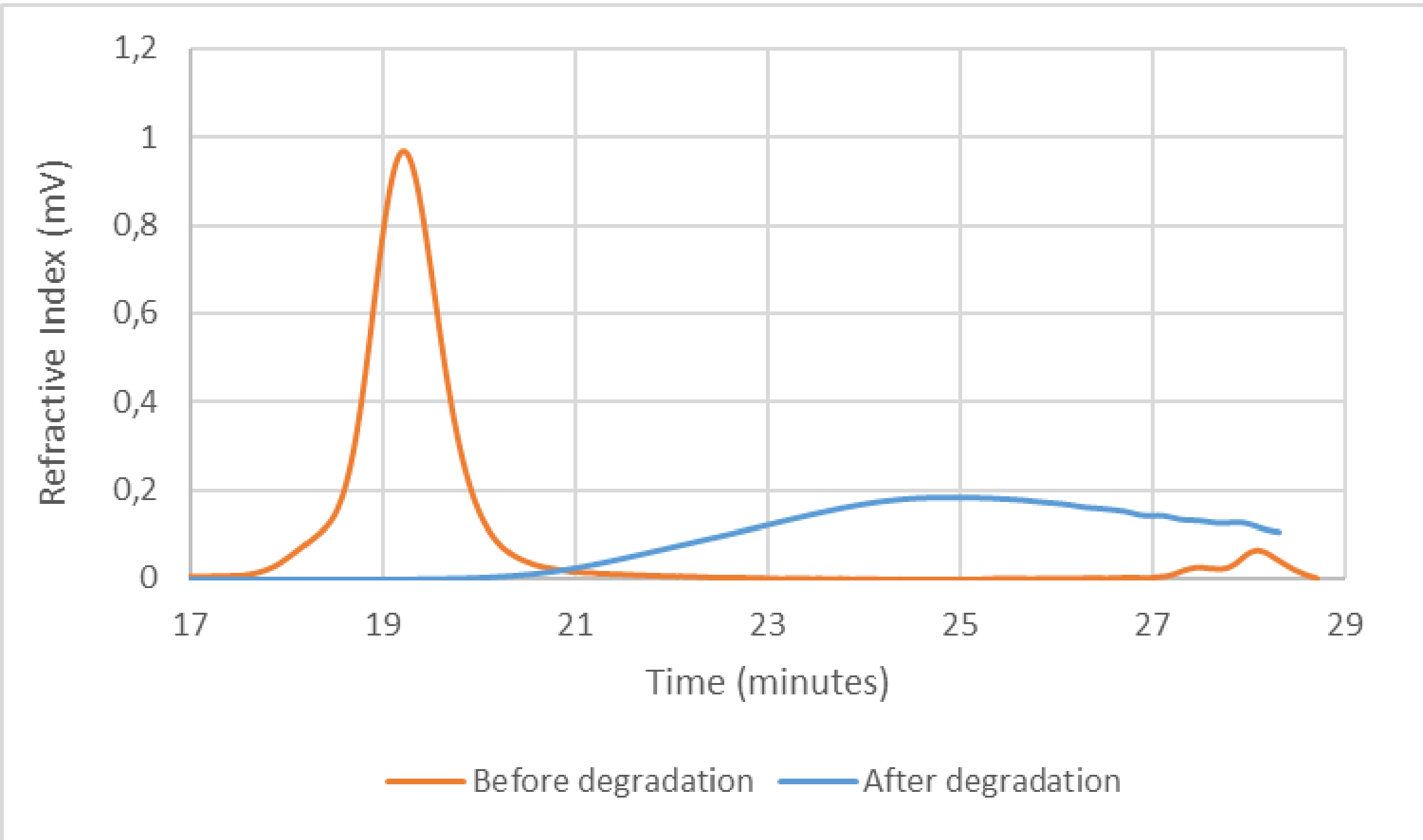

Figure S5. SEC chromatograms of CS2 before and after degradation (entry 14).

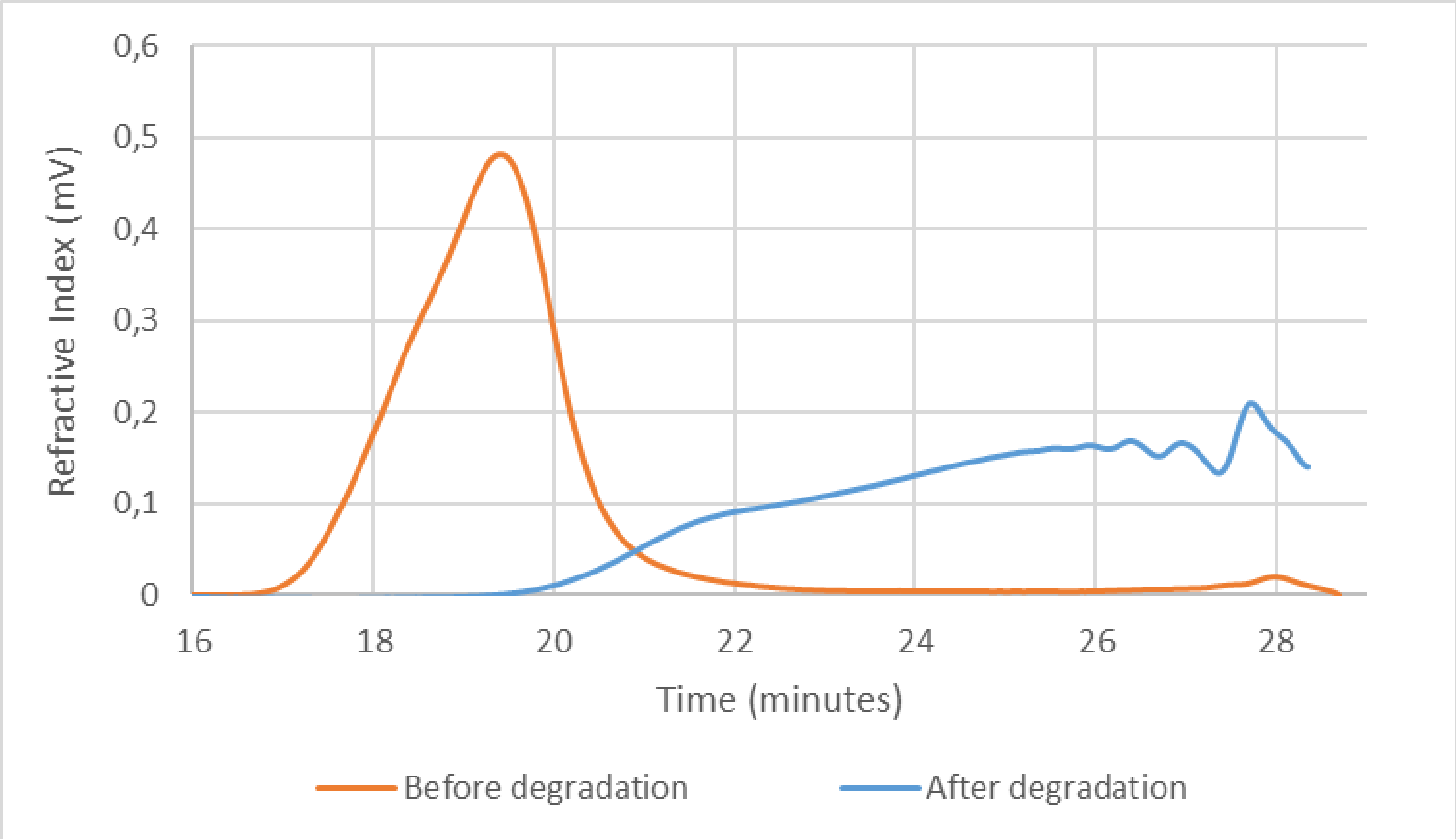

Figure S6. SEC chromatograms of CS3 before and after degradation (entry 15).

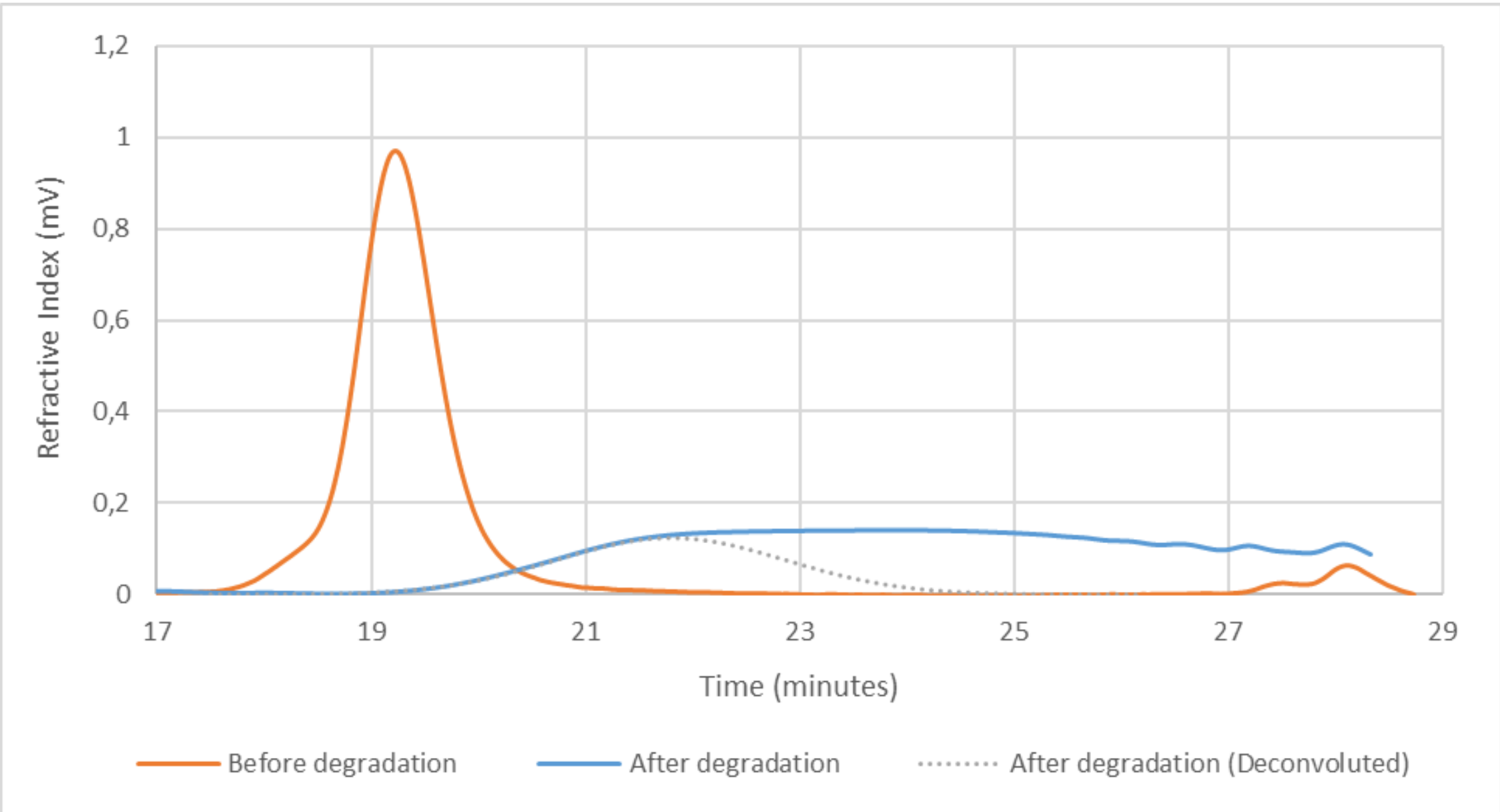

Figure S7. SEC chromatograms of CS2 before degradation and after degradation (with and without deconvolution) (entry 16).

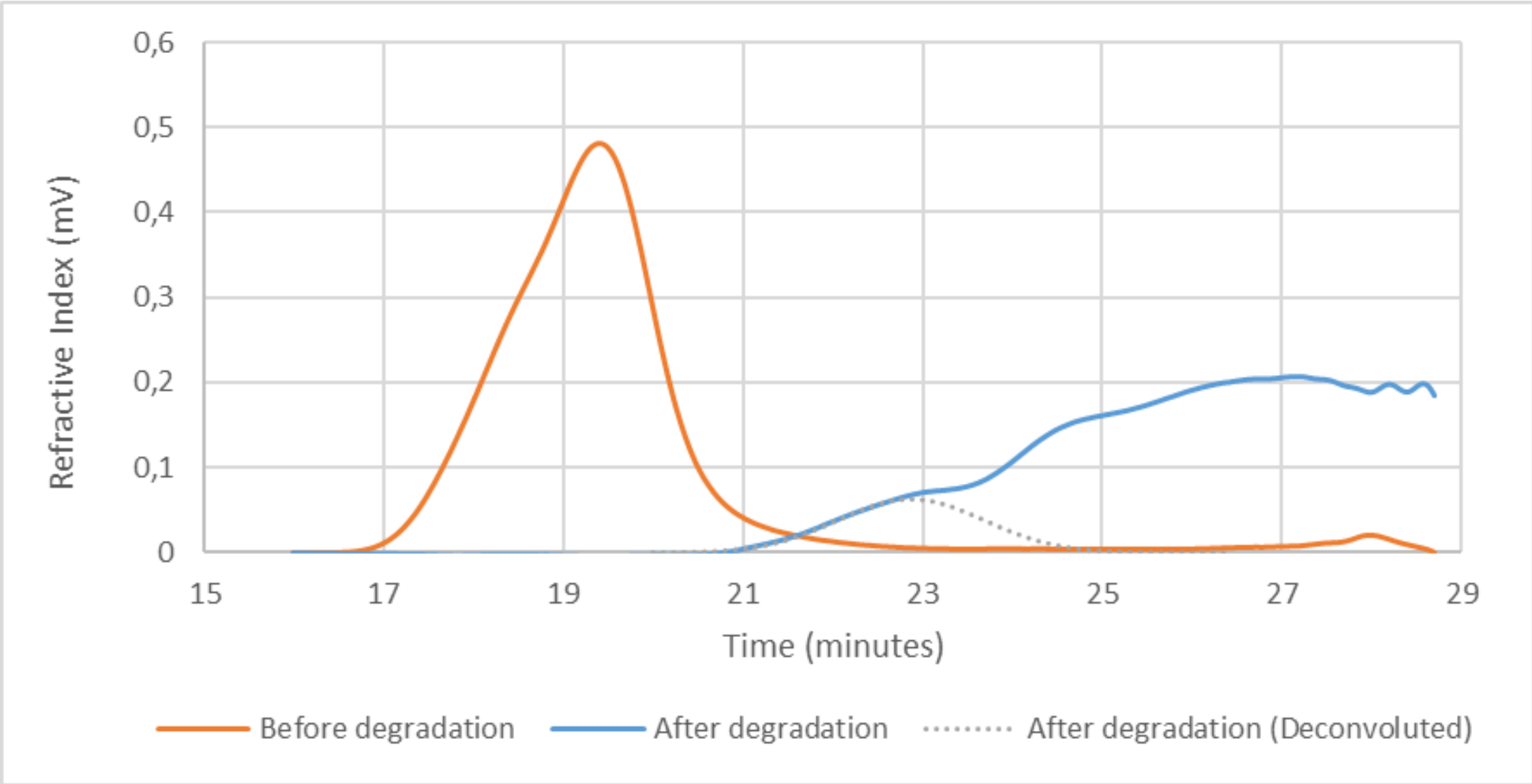

Figure S8. SEC chromatograms of CS3 before degradation and after degradation (with and without deconvolution) (entry 17).

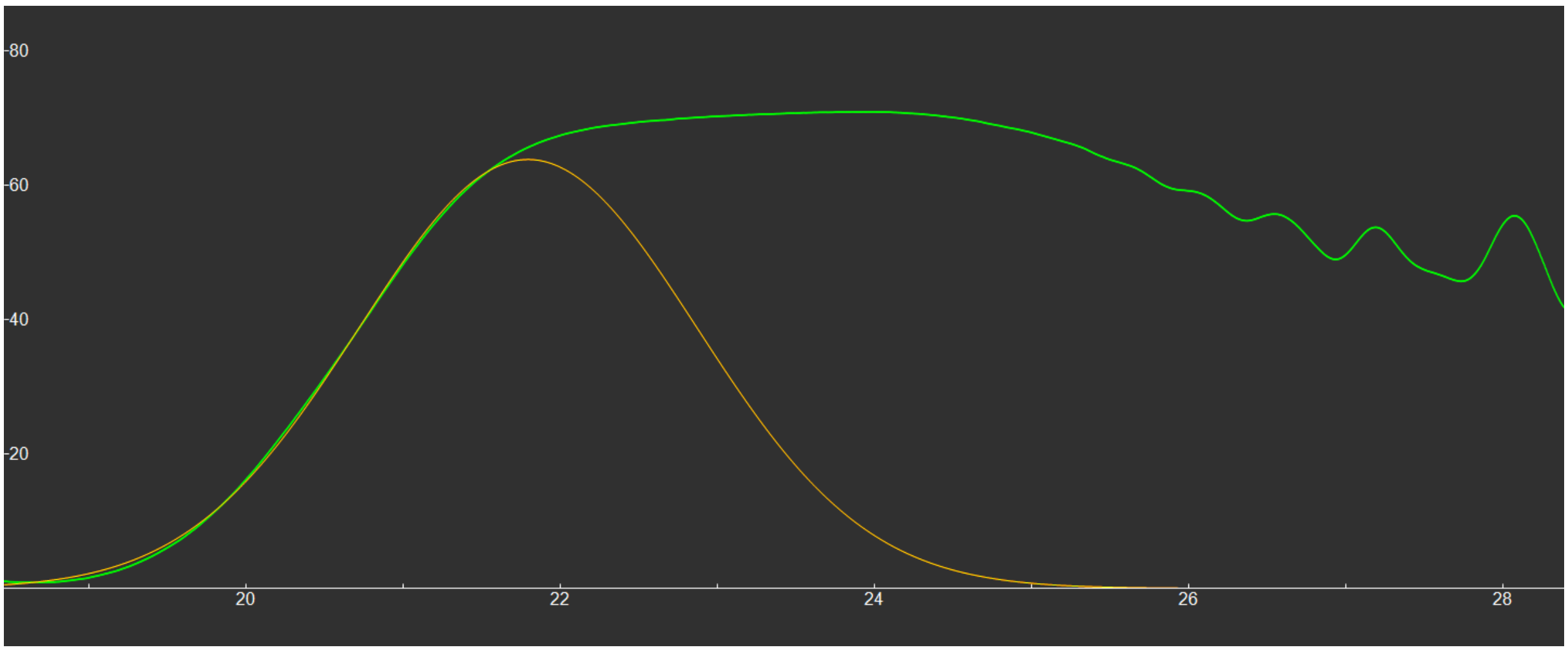

Figure S9. SEC chromatogram deconvolution after degradation: CS2 (Entry 16, Fityk Gaussian fitting).

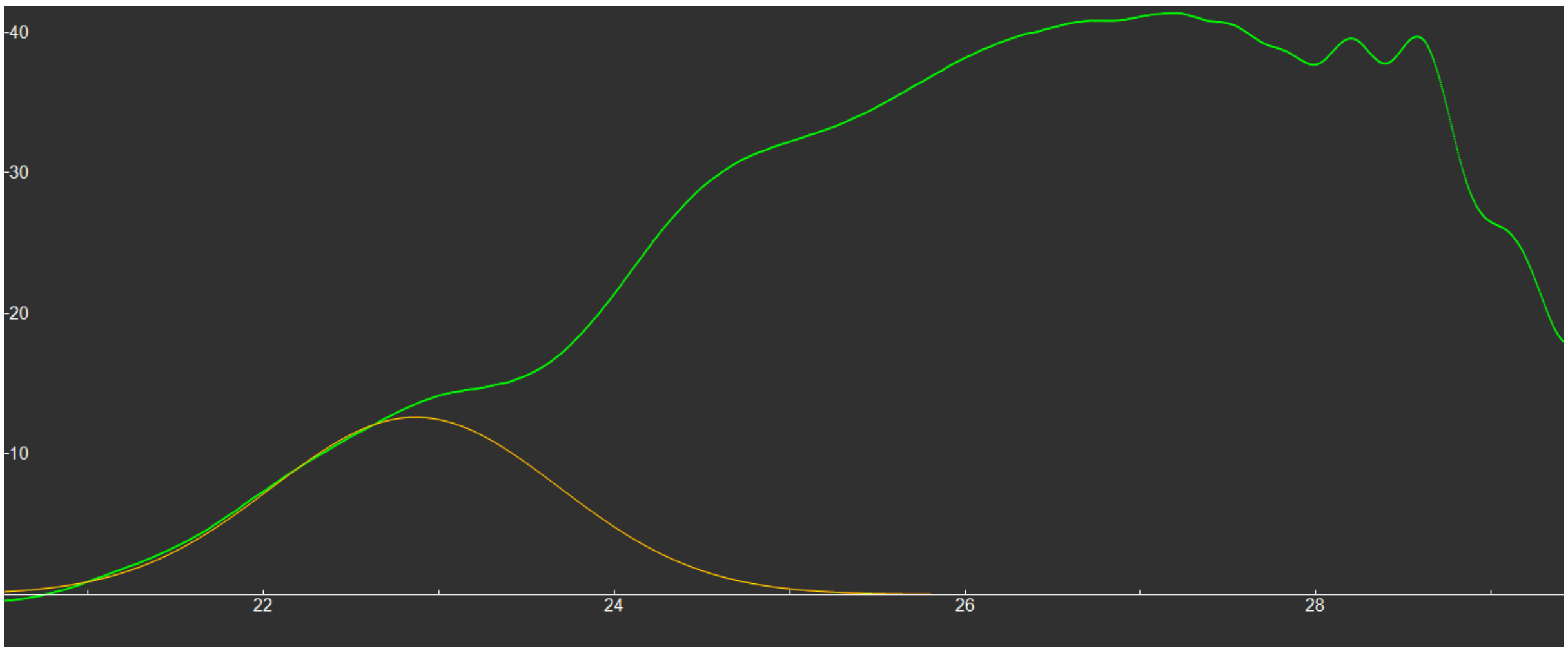

Figure S10. SEC chromatogram deconvolution after degradation: CS3 (Entry 17, Fityk Gaussian fitting).

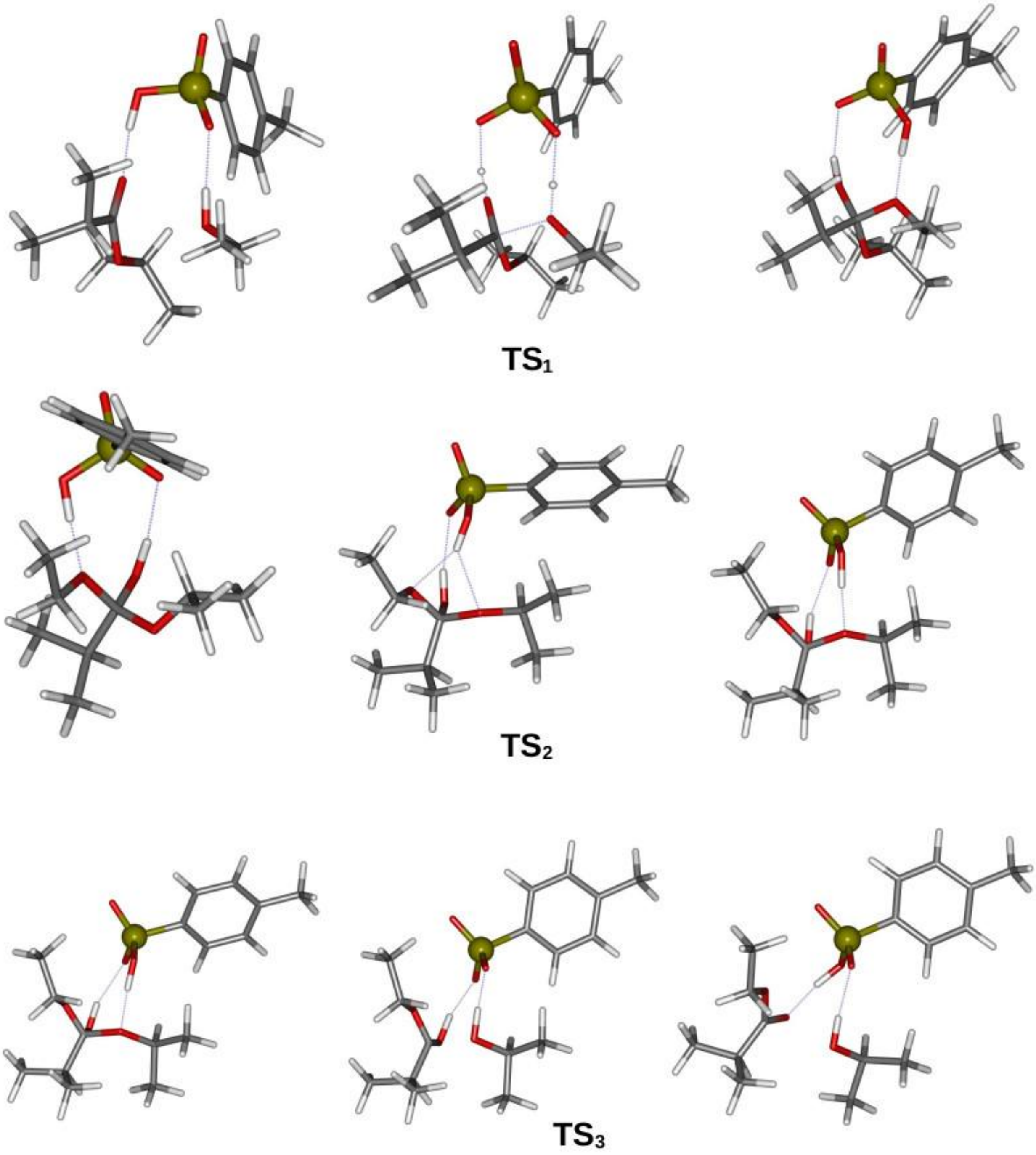

Figure S11. Optimized geometry structures of the species involved in the mechanism for the pTSA catalysed PLA reaction Color code : C grey, O red, S yellow and H white.

Enzymatic degradation of statistical copolymer with high PLLA content

Sample	Composition		M_n	Dispersity (Đ)
	PLLA	PCL	g/mol	
SS2	68%	32%	47,000	1.1

The enzymatic degradation experiment was performed on statistical copolymer SS2 (68% PLLA) under conditions reported by Vert et al[4]., namely for 3 days at 37 °C, using 1 mg of lipase at pH 7.6. After enzymatic treatment, the SEC chromatogram remained largely unchanged compared with that of the initial copolymer (Figure S12). The corresponding change in molecular weight distribution indicates only approximately 2% degradation, demonstrating that no significant enzymatic degradation occurred under these conditions.

These results are consistent with the findings reported by Vert et al., who observed limited enzymatic degradation for statistical copolymers with high PLLA content. The present results therefore further support the reported dependence of enzymatic degradation on the PLLA composition of the statistical copolymer.

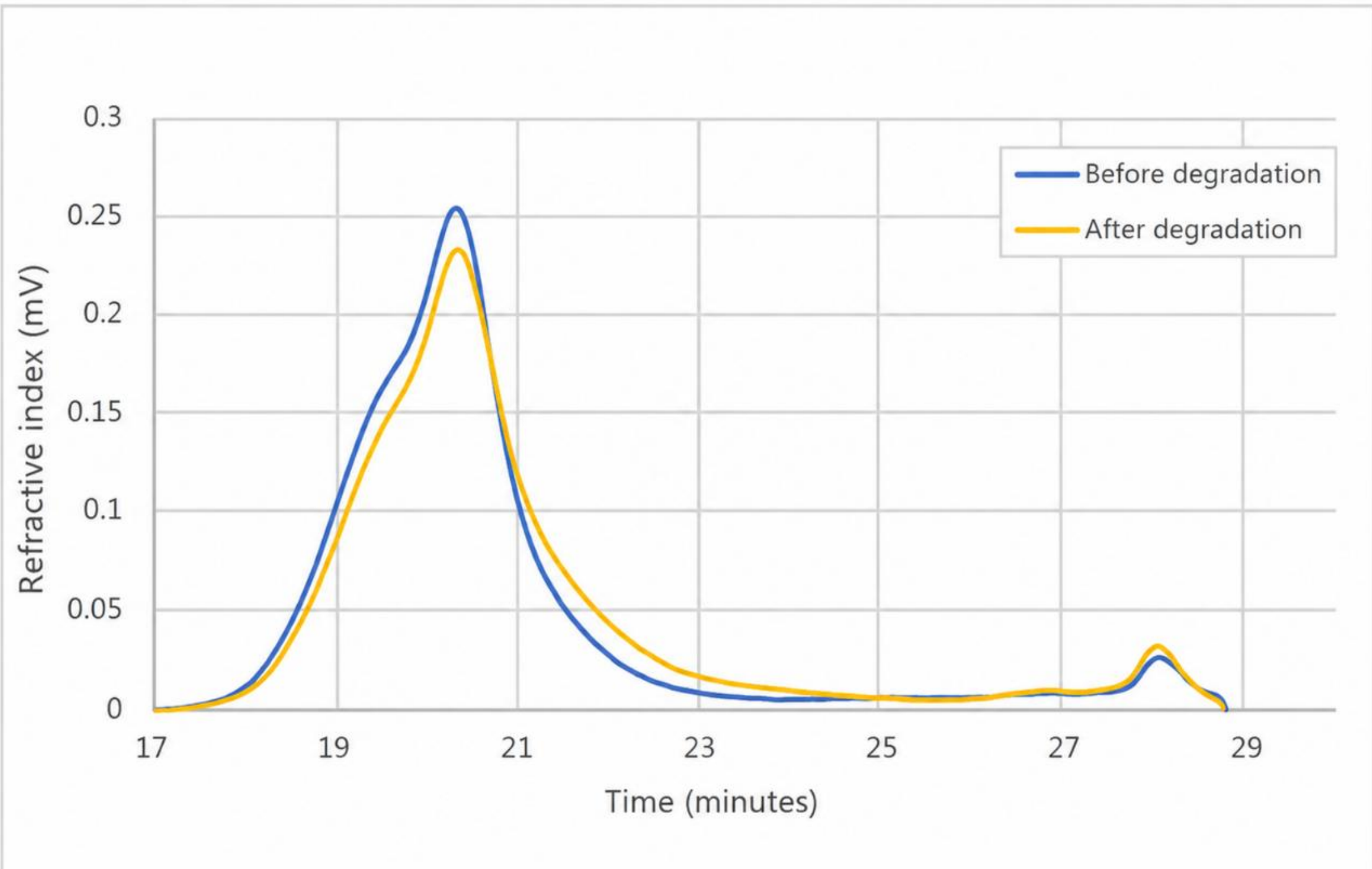

Figure S12. SEC chromatograms of SS2 before degradation and after degradation.

[4] S. Lenglet, S. Li, and M. Vert, "Lipase-catalysed degradation of copolymers prepared from ε-caprolactone and dl-lactide," Polym. Degrad. Stab., vol. 94, no. 4, Apr. 2009, doi: 10.1016/j.polymdegradstab.2008.12.023.

Blank experiments

Blank experiments were conducted on the sample CS3 under both the Brønsted base and Brønsted acid catalysed degradation conditions, but in the absence of the corresponding catalyst. For the Brønsted base conditions, the experiment was performed for 2 days at 38 °C using ethanol (0.5 equiv. relative to the ester groups). For the Brønsted acid conditions, the experiment was performed for 6 h at 60 °C using ethanol (1 equiv. relative to the ester groups).

These experiments were performed to verify that the observed polymer degradation is primarily driven by the presence of the catalyst rather than by the reaction conditions alone. As shown by the SEC chromatograms (Figures S13 and S14), the molecular weight distribution remained largely unchanged after treatment under both sets of blank conditions. Only limited degradations were observed in the absence of the catalyst, which are significantly lower than that observed under the corresponding catalyzed conditions. These results therefore support the catalytic nature of the degradation process.

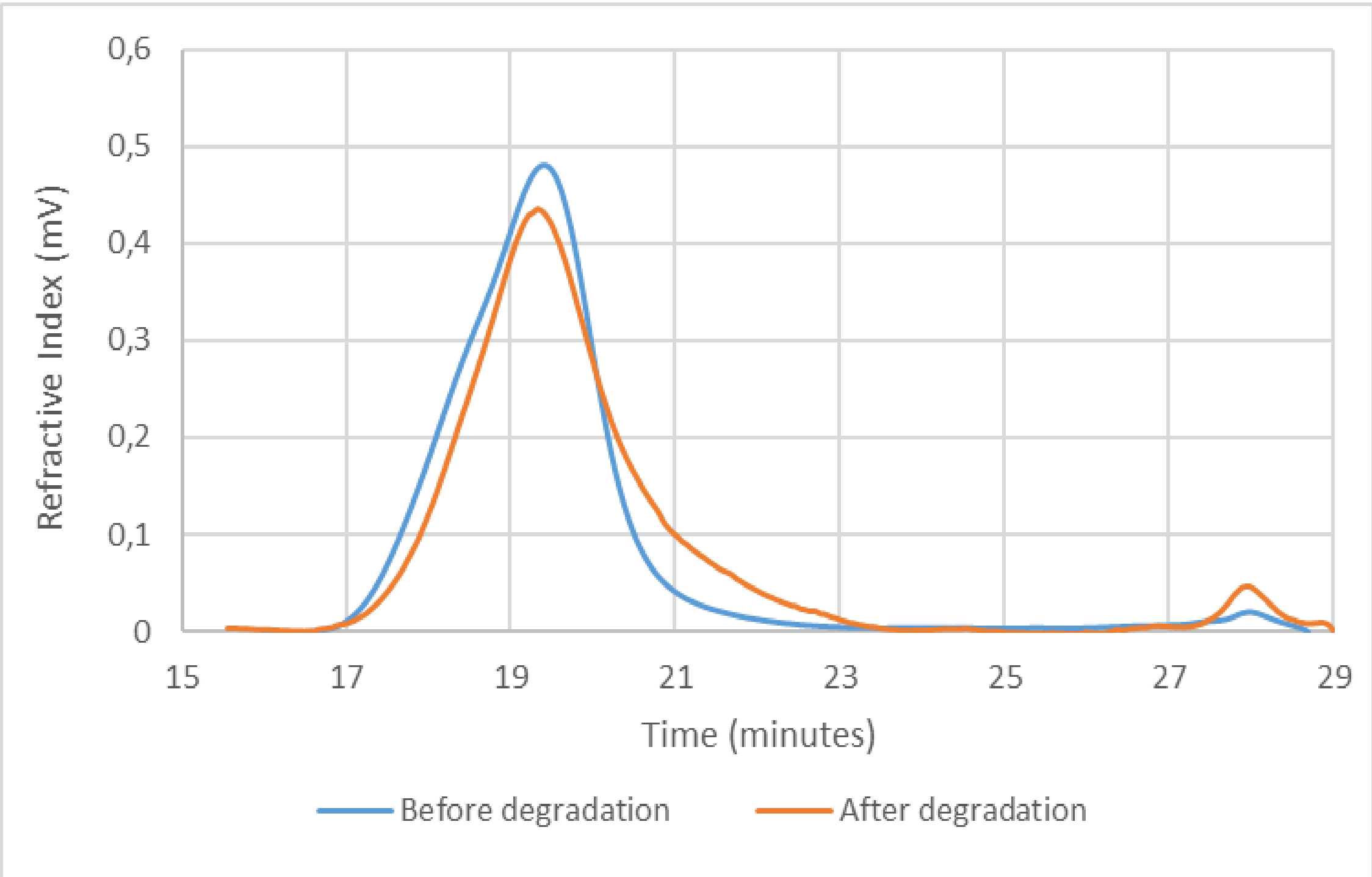

Figure S13. SEC chromatograms of CS3 before degradation and after degradation - Brønsted base blank.

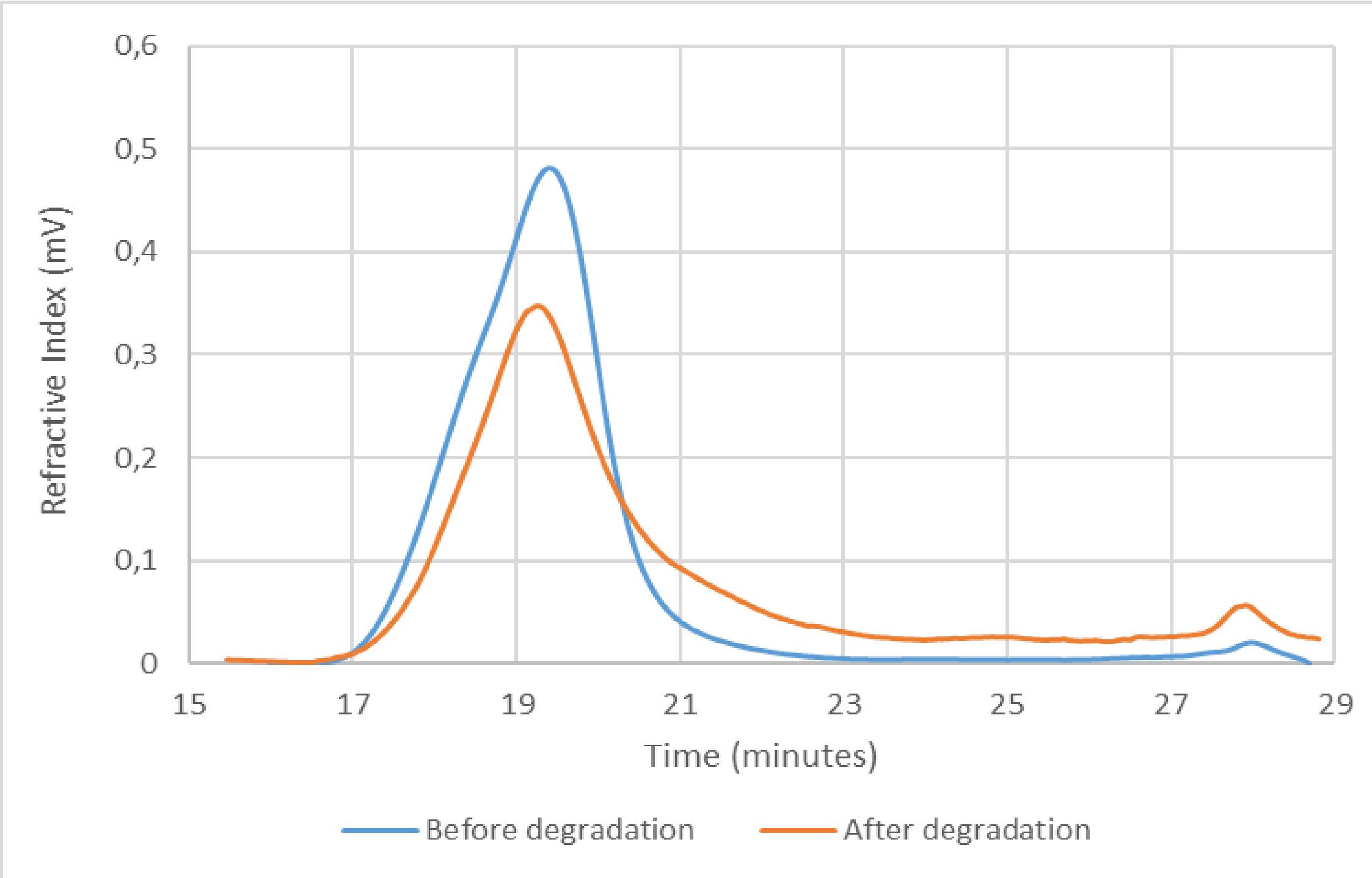

Figure S14. SEC chromatograms of CS3 before degradation and after degradation - Brønsted acid blank.

Duplicate Degradation Experiments

To assess the reproducibility of the degradation experiments, duplicate experiments were performed using the same chain shuttled copolymer sample, CS3. Enzymatic, Brønsted acid catalysed, and Brønsted base catalysed degradation experiments were independently repeated under identical reaction conditions. The results obtained from the duplicate experiments were compared to evaluate the reproducibility of the degradation behavior under each set of conditions.

Reproducibility of Enzymatic Degradation

To assess the reproducibility of the enzymatic degradation, two independent experiments (E1 and E2) were performed on the same chain shuttled copolymer sample, CS3, under identical conditions: 5 days at 37 °C using 1 g of lipase from *Pseudomonas cepacia* in a buffer at pH 7.6. Both experiments exhibited similar degradation behavior, with a degradation yield of approximately 5%. Moreover, the SEC traces obtained after degradation were nearly identical for E1 and E2 (Figure S15), further demonstrating the reproducibility of the enzymatic degradation under the investigated conditions.

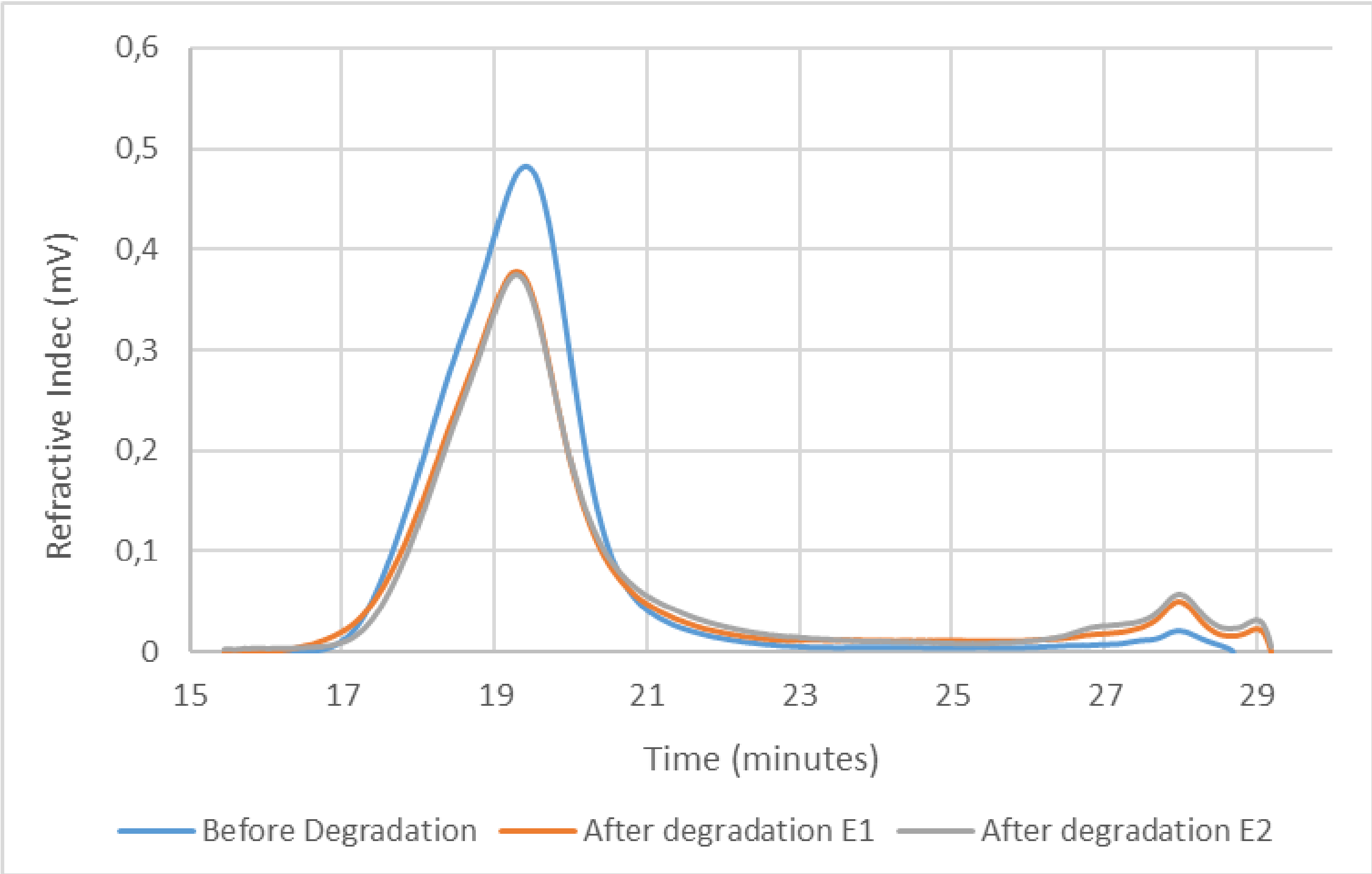

Figure S15. SEC chromatograms of CS3 before degradation and after degradation – Enzymatic duplicates.

Reproducibility of Brønsted Base Catalyzed Degradation

Two independent experiments (B1 and B2) were performed on the same chain shuttled copolymer sample, CS3, under identical degradation conditions: 2 days at 38 °C, using ethanol (0.5 mol equiv relative to the ester groups) as the degrading agent and DMAP (0.2 mol equiv relative to the ester groups) as the Brønsted base catalyst, with DCM as the solvent. In both experiments, the formation of the PLLA chain end signal at approximately 4.35 ppm was observed after degradation (Figure S16), indicating the selective degradation of the lactyl units. Moreover, the SEC traces obtained after degradation were comparable for B1 and B2 (Figure S17), further demonstrating the reproducibility of the Brønsted base catalyzed degradation under the investigated conditions.

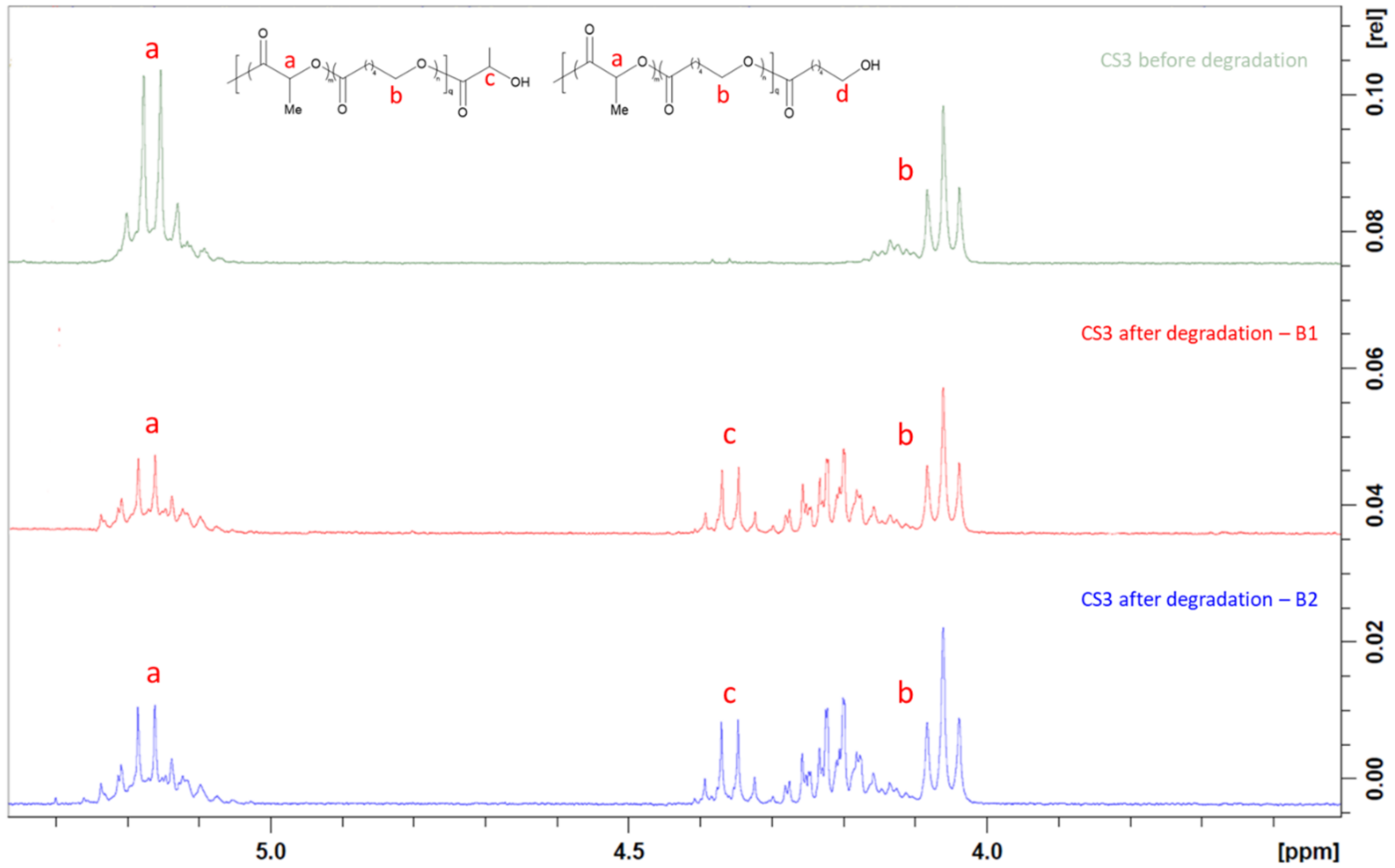

Figure S16. NMR spectra of CS3 before degradation and after degradation – Brønsted base duplicates.

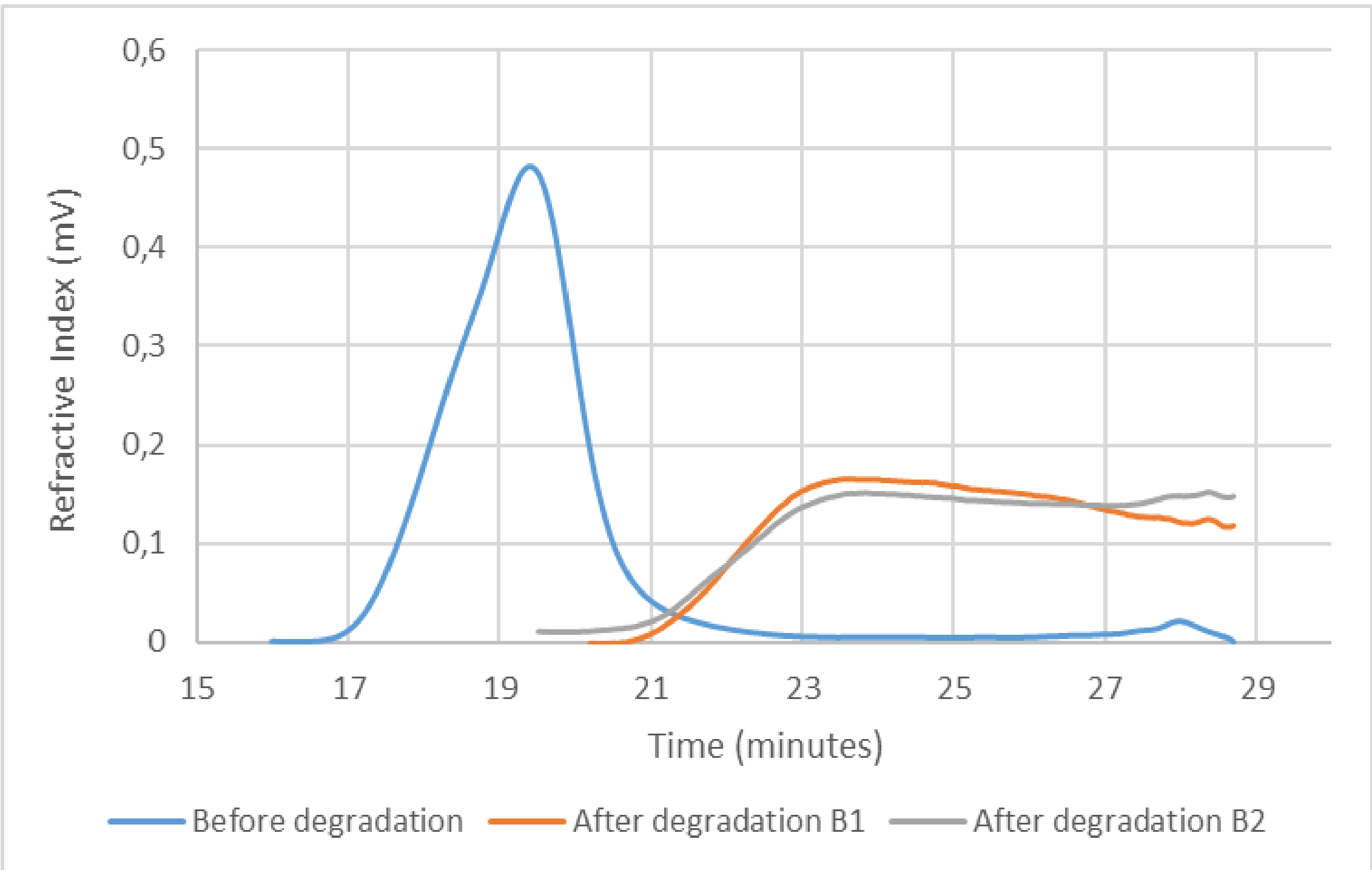

Figure S17. SEC chromatograms of CS3 before degradation and after degradation – Brønsted base duplicates.

Reproducibility of Brønsted Acid-Catalyzed Degradation

Two independent experiments (A1 and A2) were performed on the same chain shuttled copolymer sample, CS3, under identical degradation conditions: 6 h at 60 °C, using ethanol (1 mol equiv relative to the ester groups) as the degrading agent and pTSA (1/40 mol equiv relative to the monomeric units) as the catalyst, with toluene as the solvent. In both experiments, signals corresponding to the caprolactoyl units at approximately 3.65 ppm and the lactyl units at approximately 4.35 ppm were observed after degradation (Figure S18), indicating the degradation of both types of units. Quantification of the degradation revealed a preferential degradation of the caprolactoyl units in both experiments, with selectivities of 65% and 64% for A1 and A2, respectively. The close agreement between these values demonstrates the reproducibility of the observed degradation selectivity. Furthermore, comparable SEC traces were obtained after degradation for A1 and A2 (Figure S19), further supporting the reproducibility of the Brønsted acid catalyzed degradation under the investigated conditions.

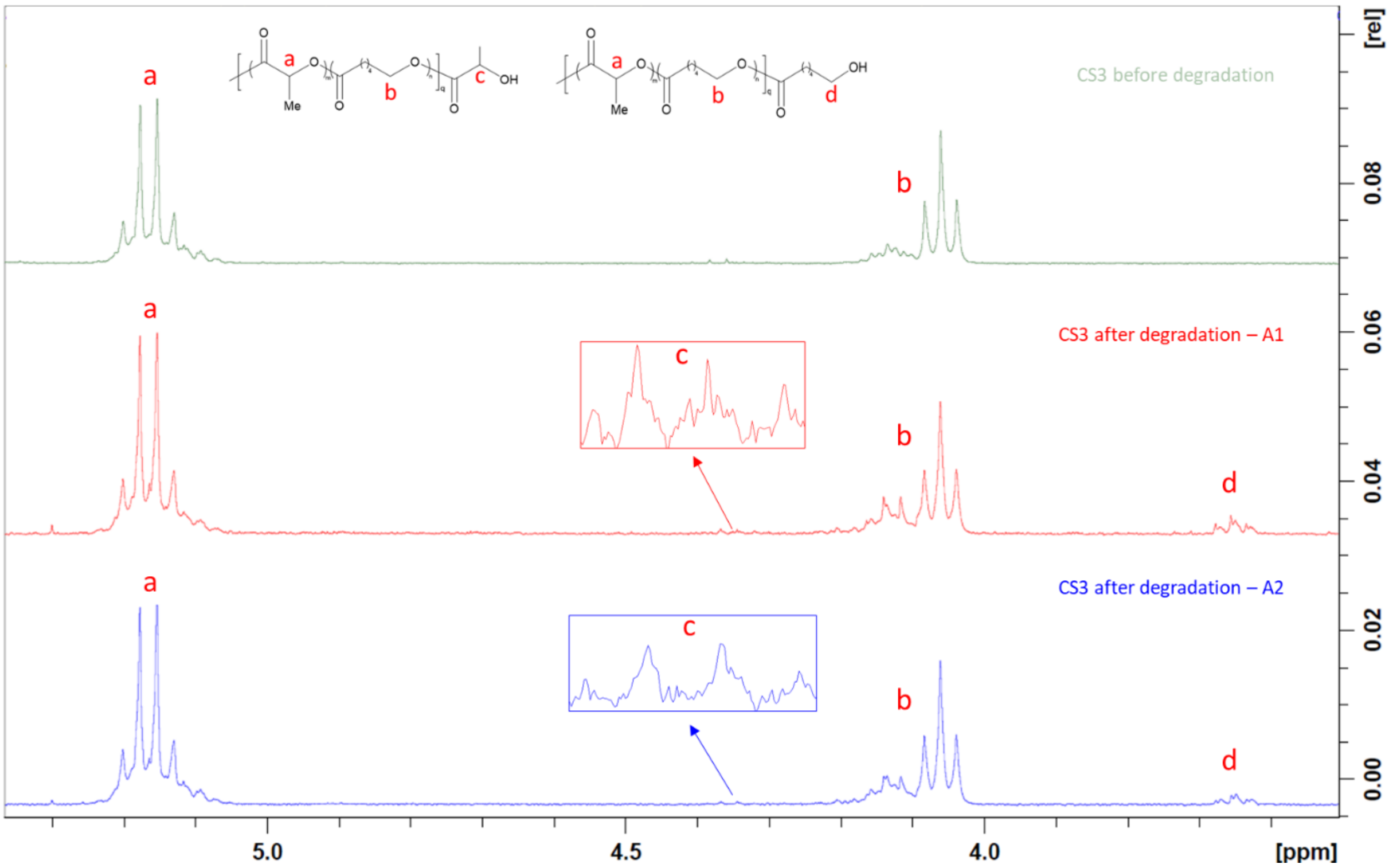

Figure S18. NMR spectra of CS3 before degradation and after degradation – Brønsted acid duplicates.

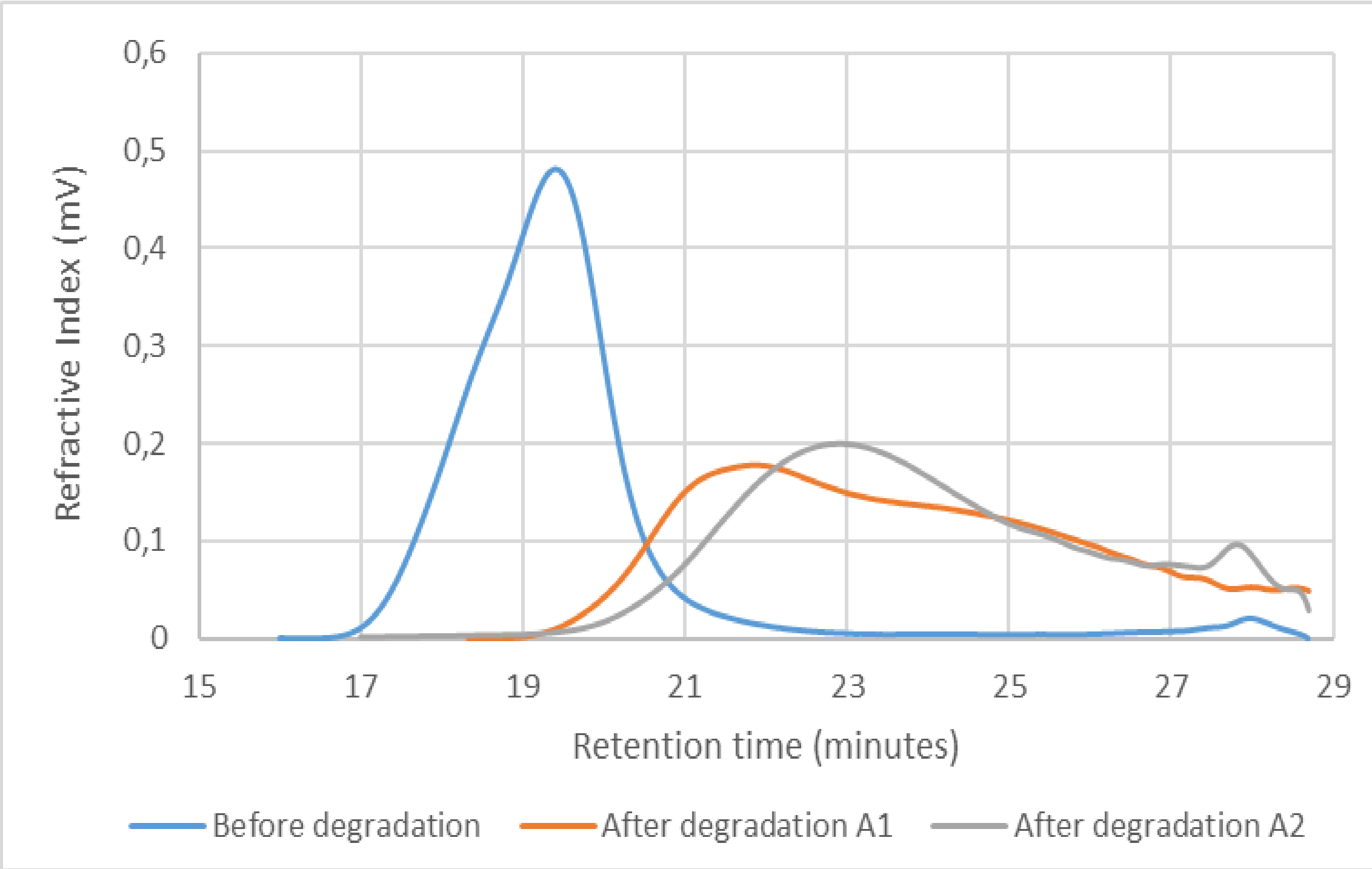

Figure S19. SEC chromatograms of CS3 before degradation and after degradation – Brønsted acid duplicates.
